# Establishing ^18^O‑Labeled Inositol Phosphates for Quantitative Capillary Electrophoresis-Mass Spectrometry: Fragmentation Pathways and Comparison with ^13^C‑Labeled Analogs

**DOI:** 10.1021/acs.analchem.5c05114

**Published:** 2025-11-05

**Authors:** Guizhen Liu, Tobias Dürr-Mayer, Mengsi Lu, Henning J. Jessen

**Affiliations:** † Institute of Organic Chemistry, 9174 University of Freiburg, Albertstrasse 21, 79104 Freiburg, Germany; ‡ CIBSS, Centre for Integrative Biological Signaling Studies, University of Freiburg, 79104 Freiburg, Germany

## Abstract

Capillary electrophoresis mass spectrometry (CE-MS) allows for the rapid and accurate quantitative analysis of inositol phosphates (InsPs) and inositol pyrophosphates (PP-InsPs). The recent discovery of new InsPs and PP-InsPs isomers in plants and mammals necessitates new heavy isotope references for quantitative analysis of complex cellular extracts. Here, we evaluate ^18^O-labeled InsPs and PP-InsPs as alternatives to ^13^C labeled internal standards for quantitation by CE-MS. In contrast to ^13^C labels, the ^18^O labels are introduced at the end of a synthetic campaign and not at the beginning, rendering ^18^O much more accessible and affordable as a label. A series of ^18^O-labeled InsPs and PP-InsPs with different numbers and positions of ^18^O atoms were synthesized, enabling systematic investigation of MS2 fragmentation pathways. We propose two major dissociation pathways to elucidate the ^18^O redistribution of the dominant product ion (the loss of H_3_PO_4_). Based on these insights, we identified the loss of HPO_3_ as a suitable transition for minimizing isotope redistribution in MS2 analysis. The ratios of this alternative product ion and dominant product ion were reproducible across replicates, concentration, and measurement days, supporting the use of this alternative product ion as a reliable product ion for quantitative analysis. Application to *Saccharomyces cerevisiae*, HCT116 cells, and *Arabidopsis thaliana* extracts confirmed accurate quantitation and precision comparable to ^13^C-based methods.

## Graphical abstract

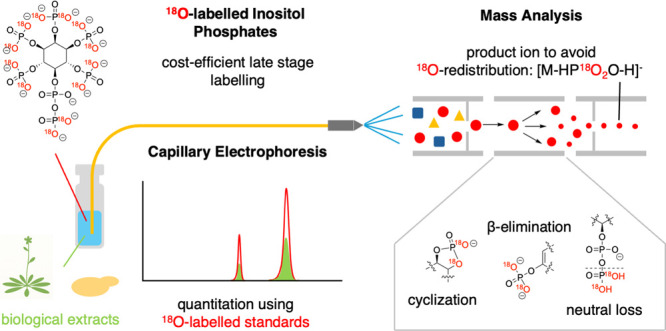

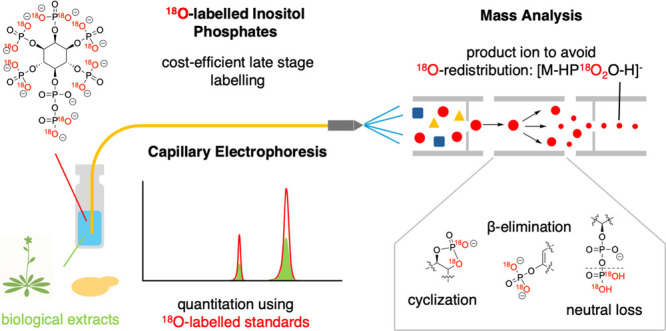

## 1 Introduction

The comprehensive analysis of inositol phosphates (InsPs) and inositol pyrophosphate (PP-InsPs, also referred to as X-InsP_7_ and X, Y-InsP_8_ with the first number indicating the position(s) of the diphosphate) from different biological species has been a technical challenge for a long time.^1–4^ The ideal analytical platform should be able to analyze different isomers of InsPs and PP-InsPs with good resolution and good sensitivity in extracts of biological samples. Capillary electrophoresis mass spectrometry (CE-MS) has proven to be a powerful tool for InsPs and PP-InsPs metabolism analysis in the past 5 years.^5–11^

One key aspect of the comprehensive analysis of InsPs and PP-InsPs involves an accurate quantitative analysis of the corresponding isomers. Previously, this either involves accurate mass in the full scan mode by QTOF or mass spectrometry fragments in the multiple reaction monitoring (MRM) mode to get better sensitivity.^8,12,13^ Chemoenzymatically synthesized ^13^C-labeled InsPs and PP-InsPs were applied as internal standards for the quantitative analysis of InsPs and PP-InsPs in complex biological samples.^8,14–16^ Additionally, with the analytical advances of methods (e.g., MS and NMR) for InsPs and PP-InsPs analysis, more understudied isomers, such as an unknown InsP_8_ isomer,^17^ 4 or 6 or 2-InsP_7_,^7,18,19^ *scyllo*-InsP_6_,^20^ Ins­(1,2,3)­P_3_ and Ins­(1,2,6)­P_3_,^6,21–23^ Ins­(2,3)­P_2_,^16,24^ and Ins(2)­P,^16,24^ were identified in biological samples.^2^ These methods aided the discovery and elucidation of the functions of new isomers in different biological species.^2,17,18,20,25–29^ However, without stable isotope labeled standards, the assignment and accurate quantitative analysis of these isomers is difficult to achieve. Therefore, pure and well-defined isotopic standards of InsPs and PP-InsPs isomers are important in the field as they enable precise identification of these molecules and facilitate the study of their metabolic pathways and biological functions.^8,15,30,31^

One very efficient methodology for the synthesis of stable isotope labeled InsPs and PP-InsPs involves a chemoenzymatic approach.^14,30,32^ Starting from ^13^C-labeled glucose, an enzymatic cascade rearranges it to ^13^C-labeled *myo*-inositol. A follow-up chemoenzymatic synthesis delivers ^13^C-labeled InsP_3_, InsP_5_, InsP_6_, InsP_7_, and InsP_8_. This enzymatic approach is effective for the scalable synthesis of well-documented InsPs and PP-InsPs, where the enzymes are available and the exact products are known. Its applicability is constrained by the availability and substrate specificity of the InsP and PP-InsP kinases. In other words, for newly discovered isomers where the enzymes are unknown, a chemical synthesis is required. This process will be costly because a significant portion of the ^13^C-labeled inositol will be lost during multiple organic transformations, which typically do not proceed in quantitative yields.

^18^O-labeling of InsP and PP-InsP standards could be an interesting alternative due to the lower cost of the ^18^O label as ^18^O water is comparably cheap and because the ^18^O labels are introduced at the end of the chemical synthesis. A direct synthesis of ^18^O-labeled InsPs and PP-InsPs was demonstrated in previous studies using modified ^18^O_2_-labeled phosphoramidite reagents, achieving good yields on a gram scale.^33–35^ With this method, it is possible to follow previously established selective syntheses of InsPs and PP-InsPs to access ^18^O-labeled variants.^36–39^ In addition, ^18^O-labeled phosphate is a useful tool to study phosphate turnover^5^ and reaction mechanisms.^40–42^

Our previous study revealed an ^18^O redistribution in 5-InsP_7_ with two ^18^O labels in the β-phosphate of the P-anhydride during MS/MS fragmentation using electrospray ionization (ESI),^5^ which limited its applicability in quantitative MS. To transform these and related isotope labeled molecules into valuable tools for quantitative MS beyond structural validation, we investigated the MS/MS fragmentation patterns of diverse synthetic ^18^O-labeled InsPs and PP-InsPs with different numbers of ^18^O labels. We propose two competing fragmentation pathways that are responsible for the observed ^18^O redistribution. Based on these insights, we optimized MS parameters and MRM transitions, enabling the reliable use of ^18^O-labeled InsPs and PP-InsPs for quantitative MS analysis as a complementation to ^13^C labeled internal references.

## 2 Materials and Methods

### 2.1 Chemicals and Reagents

Acetonitrile (MeCN), sodium bicarbonate (NaHCO_3_), dimethylformamide (DMF), and 1,8-diazabicyclo(5.4.0)­undec-7-ene (DBU) were purchased from Sigma. 5-(Ethylthio)-1H-tetrazole (ETT) was purchased from ChemPUR. Trifluoroacetic acid (TFA), *meta*-chloroperbenzoic acid (mCPBA), and magnesium sulfate (MgSO_4_) were purchased from Fisher Scientific. Triethylammonium acetate buffer pH 7.0 (1 M) was purchased from PanReac AppliChem. Methanol (MeOH), ammonium acetate (LC–MS grade), 2-propanol (LC–MS grade), and ammonia solution (10%) were purchased from Carl Roth. *Myo*-Ins­(1,4,5)­P_3_ was purchased from Sichem. ^13^C_6_-labeled InsPs and PP-InsPs used in this study were kindly provided from Dorothea Fiedler (FMP Berlin).

### 2.2 CE-ESI-QqQ and CE-ESI-QTOF System

All analyses were conducted on an Agilent CE7100 capillary electrophoresis system coupled to either a QTOF (Agilent 6545, G1948B ESI source) or Agilent 6495 Triple Quadrupole (QqQ, G1958-65638 Jet Stream ESI source) equipped with a commercial CE-MS adaptor and sprayer kit (Agilent). Both systems were operated in the negative ionization mode. A bare fused silica capillary with a total length of 100 cm (50 μm internal diameter and 365 μm outer diameter) was used for CE separation. The capillary was activated by flushing with 1 M NaOH for 10 min, followed by flushing with water for 10 min. A volatile background electrolyte (35 mM ammonium acetate titrated with ammonium hydroxide to pH = 9.75) was employed for all CE-MS experiments. Before each run, the capillary was flushed with the background electrolyte for 400 s. 30 nL of the sample was injected by applying pressure (100 mbar for 15 s). MS source and scan parameters for QTOF and QqQ are summarized in Tables 1 and 2, respectively. The sheath liquid for QTOF is composed of water and 2-propanol in a ratio of 1:1 (v/v) spiked with mass references (TFA anion, [M – H]^−^, 112.9855; HP-0921, [M – H + CH_3_COOH]^−^, 980.0163). The sheath liquid for QqQ is only composed of water and isopropanol in a ratio of 1:1 (v/v). The sheath liquid flow rate into the MS is set to 10 μL/min. The ESI-MS fragmentation of InsPs and PP-InsP standards was analyzed on the CE-QTOF system. Acquisition mode targeted MS2 was employed. The targeted mass (theoretical *m*/*z*) for each compound is listed in Table S1. For the CE-QqQ system, the multiple reaction monitoring mode (MRM) was employed, and MRM transition settings are shown in Tables S3–S5. MassHunter Workstation Software (Version 10.1) was used to control the system and acquire data.

**Table 1:** Ion Source Settings and Parameters of QTOF

instrument and scan source parameters	
gas temperature	250 °C
gas flow	3 L/min
nebulizer	5 psi
Vcap	3500 V
fragmentor	100 V
skimmer	65 V
OctopoleRFPeak	750 V

acquisition mode targeted MS2	
MS scan *m*/*z* range	50–1700
MS scan rate	1 spectra/sec
MS/MS scan *m*/*z* range	50–1700
MS/MS scan rate	1 spectra/sec
isolation width MS/MS	narrow (∼1.3 amu)
using fixed collision energies	0 V, 3 V, 5 V, 10 V, 20 V, 30 V
max precursors per cycle	2
threshold	5 Abs
threshold (Rel)	0.01

**Table 2:** Ion Source Settings and Parameters of QqQ

source parameters	
gas temperature	150 °C
gas flow	11 L/min
nebulizer	8 psi
sheath gas temperature	175 °C
sheath gas flow	8 L/min
capillary voltage	–2000 V
nozzle voltage	2000 V
high-pressure RF (ion funnel parameters)	70 V
low-pressure RF (ion funnel parameters)	40 V

### 2.3 Data Analysis and Calculation

Peak intensity was extracted and collected using MassHunter Software (QqQ quantitative Analysis and Q-TOF Quantitative analysis) from RAW files. The relative abundances were calculated directly from the ion abundances of each peak. The final percentage was calculated using Microsoft Excel according to

$$relative\;abundance\;of\;pathway\;A\;\left(\%\right)=100\times \frac{peak\;area\;of\;productions\;from\;pathway\;A}{peak\;area\;of\;productions\;from\;pathways\;A\;\text{and}\;B}$$

$$relative\;abundance\;of\;pathway\;B\;\left(\%\right)=100\times \frac{peak\;area\;of\;productions\;from\;pathway\;B}{peak\;area\;of\;productions\;from\;pathways\;A\;\text{and}\;B}$$

## 3 Results and Discussion

### 3.1 ESI-MS2 Fragmentation Pattern of InsPs and PP-InsPs

We investigated the ESI MS/MS fragmentation patterns of InsPs and PP-InsPs using CE-QTOF by analyzing unlabeled and ^18^O-labeled Ins­(1,4,5)­P_3_, InsP_6_, and 5-InsP_7_. Of particular interest was the redistribution of ^18^O labels during this process, or so-called “scrambling”. The theoretical and observed abundant precursor *m*/*z* values for all species are summarized in Table S1.

#### 3.1.1 Ins­(1,4,5)­P_3_

In the negative mode, Ins­(1,4,5)­P_3_ yields abundant [M – H]^−^ ions (*m*/*z* 418.9537) by ESI. Following ESI fragmentation, the [M – H]^−^ ions yield a series of daughter ions indicated in Figure 1A. Among these, *m*/*z* 78.9591, *m*/*z* 96.9689, *m*/*z* 158.9260, and *m*/*z* 176.9353 correspond to PO_3_^–^, H_2_PO_4_^–^, HP_2_O_6_^–^, and H_3_P_2_O_7_^–^. These fragmentations are nonspecific product ions as they are also observed in the MS2 fragmentation of other InsPs and PP-InsPs. Fragment ions with *m*/*z* > 300 increase analytical specificity and were considered specific product ions. However, these product ions do not guarantee absolute structural specificity as different positional isomers can produce the same *m*/*z* value. Among them, *m*/*z* 320.9775, corresponding to [M-H_3_PO_4_-H]^−^, is the most abundant. The ion at *m*/*z* 338.9888, assigned to [M-HPO_3_-H]^−^, also has a high intensity. Under the experimental collision energy conditions, [M-H_3_PO_4_-H]^−^ is consistently the dominant product ion. Nevertheless, the relative abundance of [M-HPO_3_-H]^−^ increases with a rising collision energy (Figure 1B).

**Figure 1:**
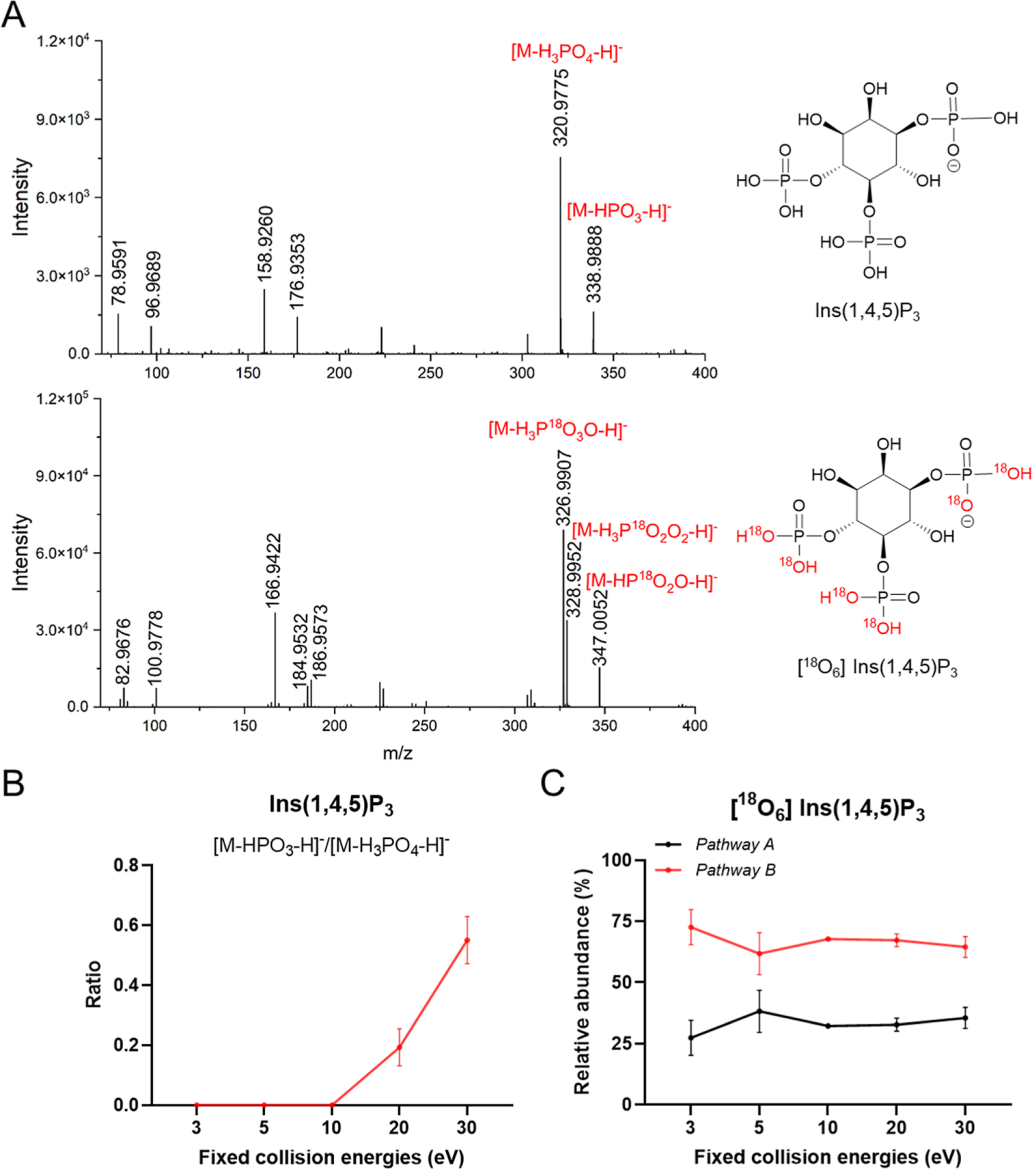
(A) MS/MS fragmentation from the singly charged precursor of Ins­(1,4,5)­P_3_ (at *m*/*z* 418.9537) and ^18^O_6_-labeled Ins­(1,4,5)­P_3_ (at *m*/*z* 430.9804) using a collision energy at 20 eV. (B) Ratio of the abundances of [M-HPO_3_-H]^−^ and [M-H_3_PO_4_-H]^−^ observed at different collision energies in the unlabeled Ins­(1,4,5)­P_3_. (C) Relative abundances of the product ions generated from pathways A and B at different collision energies in ^18^O_6_-labeled Ins­(1,4,5)­P_3_.

In comparison, ^18^O_6_-labeled Ins­(1,4,5)­P_3_, i.e., an InsP_3_ that has two nonbridging ^18^O labels in each of the three phosphate groups [M – H]^−^ (*m*/*z* 430.9804) is the most abundant precursor ion by ESI as well (M + 12 due to 6 ^18^O labels). The specific product ions generated from ESI fragmentation of ^18^O_6_-labeled Ins­(1,4,5)­P_3_ are more complex than those observed for the unlabeled Ins­(1,4,5)­P_3_. While the unlabeled Ins­(1,4,5)­P_3_ typically yields [M-H_3_PO_4_-H]^−^ (*m*/*z* 320.9775), the ^18^O_6_-labeled Ins­(1,4,5)­P_3_ produces distinct ions at [M-H_3_P^18^O_3_O-H]^−^ (*m*/*z* 326.9907) and [M-H_3_P^18^O_2_O_2_-H]^−^ (*m*/*z* 328.9950), indicating a more complex fragmentation as can be delineated for unlabeled molecules. Otherwise, the loss of 3 ^18^O atoms could not be explained. We propose a dissociation pathway for the loss of H_3_P^18^O_2_O_2_, in which a five-membered cyclic phosphate is generated initially by the attack of an adjacent phosphate group on the inositol ring, leading to the removal of the H_3_P^18^O_2_O_2_ or as the result of a β-elimination of inositol phosphates to create a C=C or C=O double bond on the ring (Scheme 1A). Regarding the unexpected loss of phosphate with a redistribution of ^18^O labels, leading to the loss of three ^18^O labels (loss of H_3_P^18^O_3_O from precursor ion), we propose pathway B (Scheme 1). This pathway involves the neutral loss of HP^18^O_2_O, yielding an abundant product ion at *m*/*z* 347.0052, followed by the subsequent loss of H_2_^18^O via cyclophosphate formation. Both pathways could occur in parallel during the fragmentation and should of course also occur for nonlabeled substrates, but here, they cannot be seen.

**Scheme 1:**
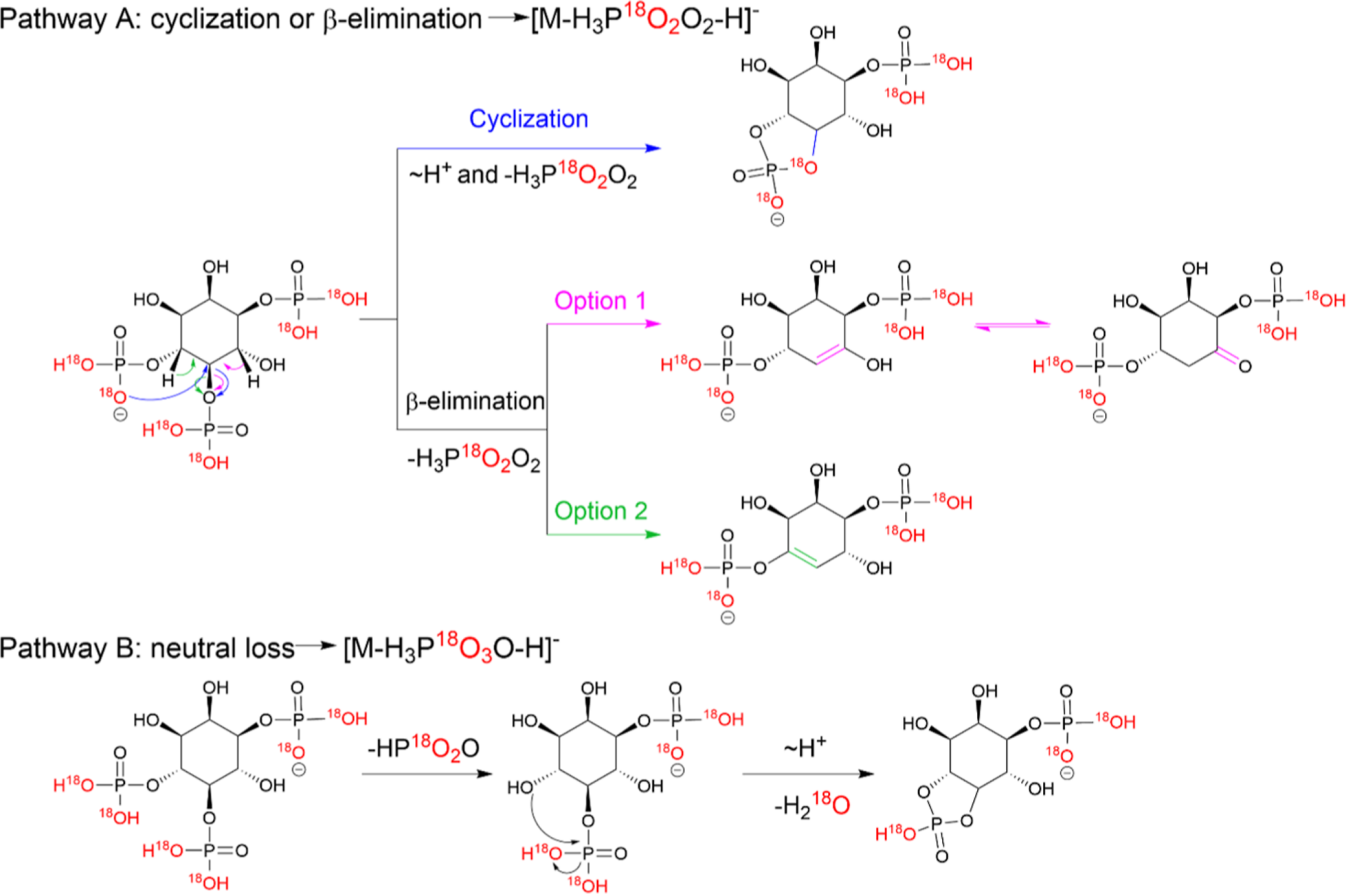
Proposed Fragmentation Pathways A and B Leading to the Product Ions [M-H_3_P^18^O_2_O_2_-H]^−^ and [M-H_3_P^18^O_3_O-H]^−^ from the Precursor Ion of ^18^O_6_-Labelled Ins­(1,4,5)­P_3_; ∼H^+^ Indicates a Proton Transfer

To further investigate the contributions of the two fragmentation pathways to product ion generation proposed in Scheme 1, the effect of the collision energy as a key factor in fragmentation was studied. In collision-cell fragmentation experiments, as the collision energy increased in the range from 0 to 20 eV, the abundance of both product ions increased (Figure S1). The ion [M-H_3_P^18^O_3_O-H]^−^ (*m*/*z* 326.9907), corresponding to pathway B, increased steeply up to 20 eV and then decreased, whereas the ion [M-H_3_P^18^O_2_O_2_-H]^−^ (*m*/*z* 328.9950) from pathway A showed a less pronounced increase from 0 to 20 eV and then decreased. When collision energy was set to 40 eV, the intensity of [M-H_3_PO_4_-H]^−^ decreased by approximately 10-fold compared with that at 30 eV in the unlabeled Ins­(1,4,5)­P_3_ (Figure S2). For the ^18^O_6_-labeled Ins­(1,4,5)­P_3_, the intensity of [M-H_3_P^18^O_3_O-H]^−^ (generated by pathway B) decreased by 10-fold at 40 eV relative to 30 eV. The ion produced by pathway A, [M-H_3_P^18^O_2_O_2_-H]^−^, became undetectable, with a signal-to-noise ratio of approximately 3. To evaluate the contributions of pathways A and B, relative abundances (%) were calculated (Figure 1C). The relative abundance of each pathway was defined as the percentage of product ion abundance produced by the respective pathway relative to the total abundance of product ions from pathways A and B. As shown in Figure 1C, pathway B is the predominant fragmentation pathway, with approximately twice the relative abundance over pathway A. We also evaluated the effect of source parameters, including gas temperature and capillary voltage, on the relative abundance of the ions produced from pathways A and B. Neither gas temperature (100–250 °C) nor capillary voltage (Vcap, 2–4 kV) significantly affected the relative abundances of ions generated from pathways A and B.

#### 3.1.2 InsP_6_

Similar ions as found for InsP_3_ were observed for InsP_6_ (Figure 2A). In unlabeled InsP_6_, the most abundant precursor ion is the doubly charged species at *m*/*z* 328.9233. This ion undergoes further fragmentations to produce several specific ions, including *m*/*z* 578.8866 (corresponding to the neutral loss of HPO_3_ from its precursor ion) and *m*/*z* 480.9101 (corresponding to the sequential loss of HPO_3_ and H_3_PO_4_ from its precursor ion). Unlike InsP_3_, where [M-H_3_PO_4_-H]^−^ is consistently the dominant product ion irrespective of the conditions compared to [M-HPO_3_-H]^−^, it is less abundant than [M-HPO_3_-H]^−^ in the case of InsP_6_ (Figure S3). Instead, the dominant product ions for InsP_6_ are [M-HPO_3_-H]^−^ and [M-HPO_3_-H_3_PO_4_-H]^−^ and they vary depending on the collision energy (Figure 2B). As shown in Figure 2B, at low collision energies (3–10 eV), the intensity of [M-HPO_3_-H]^−^ is slightly higher (1.3- to 1.7-folds) than that of [M-HPO_3_-H_3_PO_4_-H]^−^. However, at collision energies of up to 20 eV, [M-HPO_3_-H_3_PO_4_-H]^−^ becomes the dominant fragment, while [M-HPO_3_-H]^−^ decreases to approximately 10% of the intensity of [M-HPO_3_-H_3_PO_4_-H]^−^.

**Figure 2:**
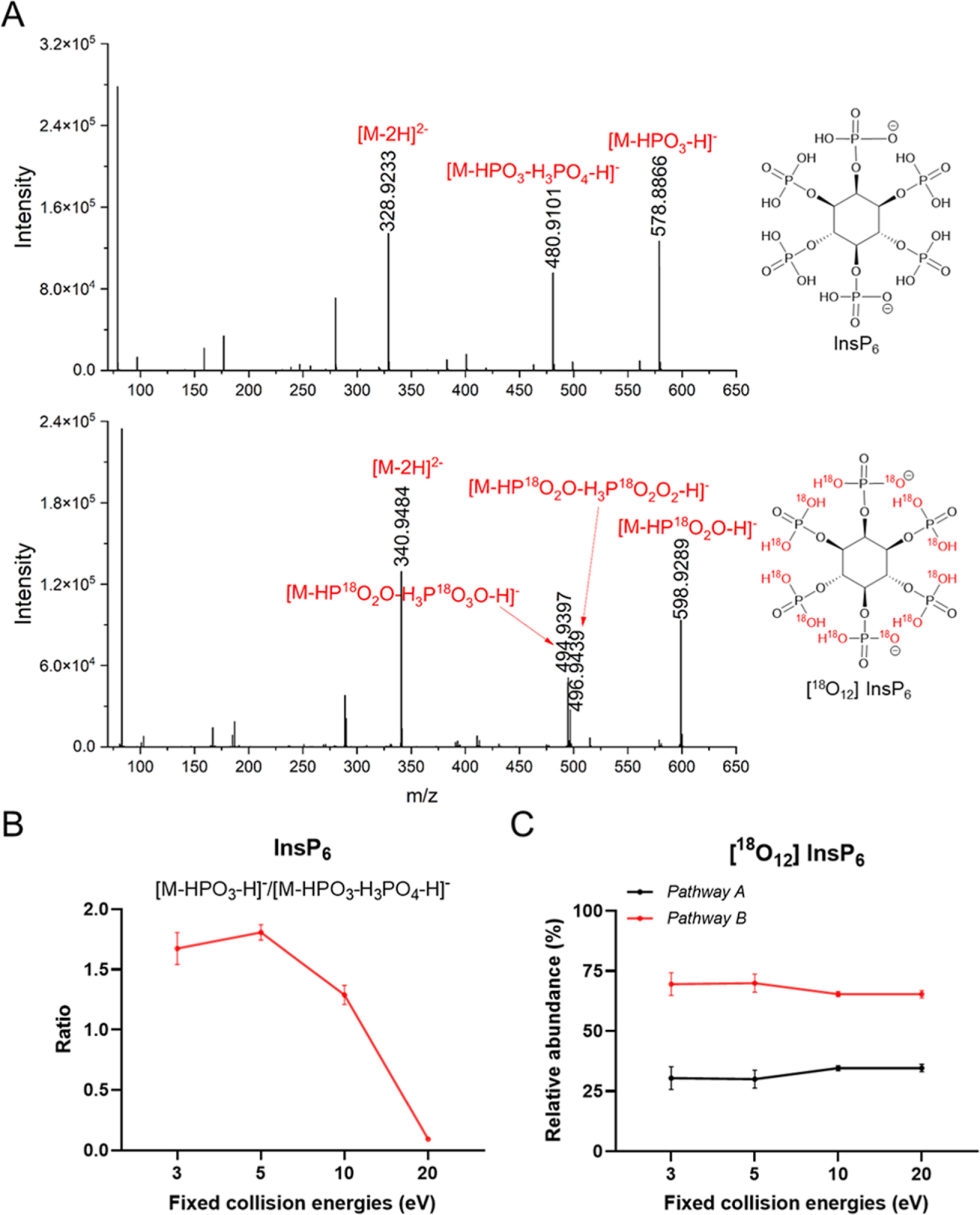
(A) MS/MS fragmentation from the doubly charged precursor of InsP_6_ (at *m*/*z* 328.9233) and ^18^O_12_-labeled InsP_6_ (at *m*/*z* 340.9484) using collision energies with 10 eV. (B) Ratio of the abundance of two major product ions [M-HPO_3_-H]^−^ and [M-HPO_3_-H_3_PO_4_-H]^−^ observed in the unlabeled InsP_6_. (C) Relative abundances of the product ions generated from pathways A and B at different collision energies in the ^18^O_12_-labeled InsP_6_.

Again, the fragmentation behavior of ^18^O_12_-labeled InsP_6_ under varying collision energies (Figure 2C) produces more product ions. Consistent with the observations for ^18^O_6_-labeled InsP_3_, ^18^O redistributions were observed in the major product ions, namely [M-HP^18^O_2_O-H_3_P^18^O_3_O-H]^−^ and [M-HP^18^O_2_O-H_3_P^18^O_2_O_2_-H]^−^, suggesting that there are two main fragmentation pathways (Scheme 2). In pathway A, the doubly charged precursor ion loses one HP^18^O_2_O and then undergoes a mechanism similar to that of ^18^O_6_-labeled InsP_3_, in which the subsequent loss of H_3_P^18^O_2_O_2_ occurs via cyclization of an adjacent phosphate or through β-elimination of inositol phosphates, creating a double bond. Protonation then yields a singly charged product ion. In pathway B, which dominates, HP^18^O_2_O is also lost initially, followed by an additional loss of HP^18^O_2_O, proton transfer, and subsequent loss of H_2_^18^O, yielding [M-HP^18^O_2_O-H_3_P^18^O_3_O-H]^−^ as the major product ion.

**Scheme 2:**
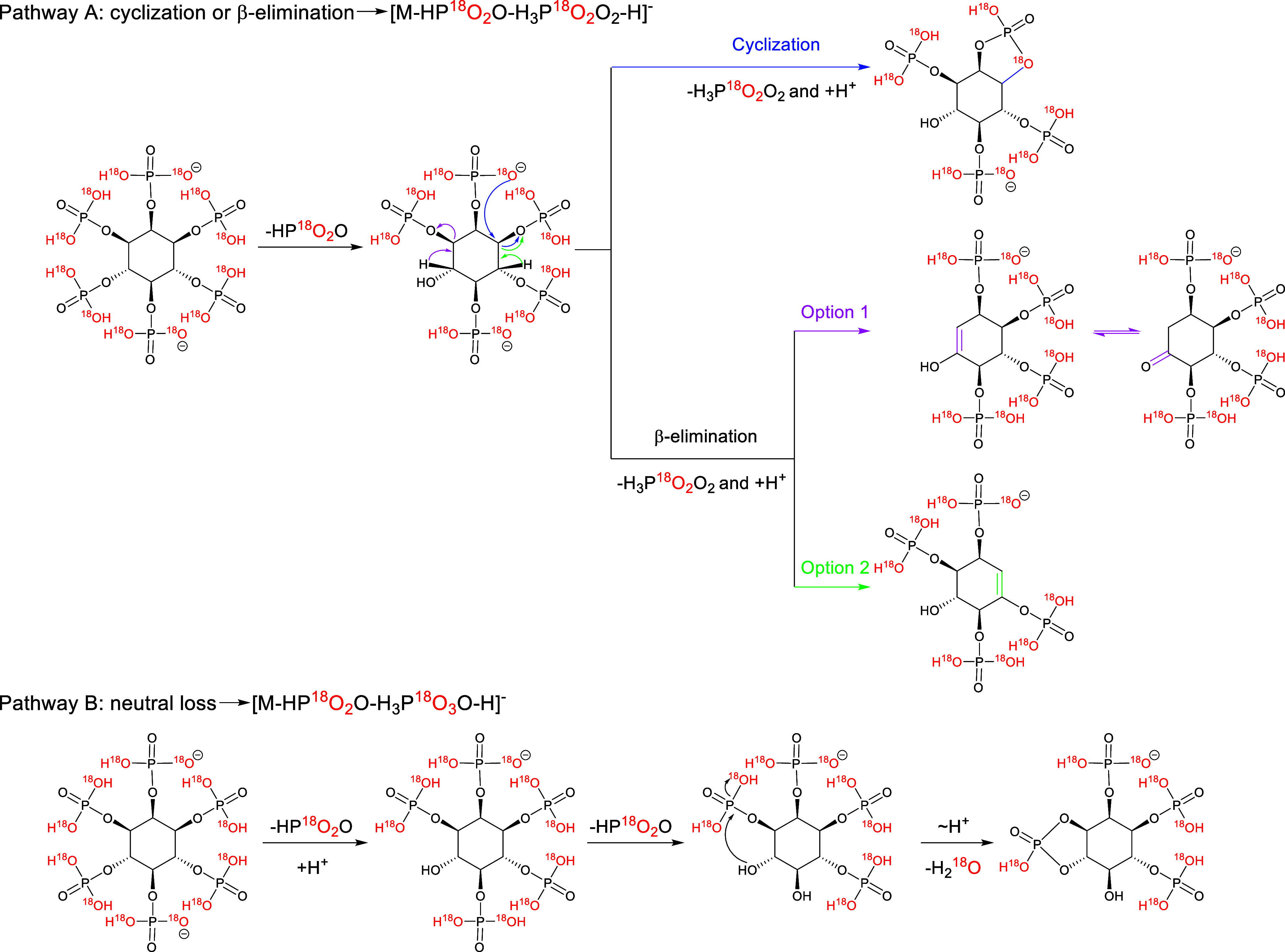
Proposed Fragmentation Pathways A and B Leading to the Product Ions [M-HP^18^O_2_O-H_3_P^18^O_2_O_2_-H]^−^ and [M-HP^18^O_2_O-H_3_P^18^O_3_O-H]^−^ from the Precursor Ion of ^18^O_12_-Labelled InsP_6_; ∼ H^+^ Indicates a Proton Transfer; + H^+^ Indicates the Gain of a Proton; The Depicted Structures Represent Only One Possible Isomer, as the Negative Charge Could Also be Located at Other Positions and Cyclizations/Eliminations at Other Positions are Conceivable, Producing Ions of the Same Mass

#### 3.1.3 5-InsP_7_

We then investigated the fragmentation behavior of inositol pyrophosphates, in particular, 5-PP-InsP_5_ (5-InsP_7_; structure shown in Figure 3). In PP-InsPs, two P–O bonds are susceptible to cleavage during ESI fragmentation: the P–O–P phosphoanhydride bond of the pyrophosphate group, which is more labile, and the C–O–P bond protruding from the inositol ring structure. As a result, ESI MS/MS typically produces fragment ions corresponding to the loss of H_3_PO_4_, yielding either singly charged [M-H_3_PO_4_-H]^−^ or doubly charged [M-H_3_PO_4_-2H]^2–^ anions. In the case of 5-InsP_7_, doubly charged ions [M-H_3_PO_4_-2H]^2–^ were observed predominantly (Figure S4). As seen with InsP_3_ and InsP_6_, [M-HPO_3_-H]^−^ was also detected. Interestingly, in the fragmentation of ^18^O_2_-labeled 5-InsP_7_ (having two ^18^O labels located at the terminal β-phosphate of the diphosphate group), both the [M-HP^18^O_2_O-H]^−^ product ion and [M-HPO_3_-H]^−^ were observed (Figure 3A). This suggests that neutral HPO_3_ loss can originate not only from the diphosphate but also from monophosphate esters, consistent with observations for the lower InsPs: InsP_3_ and InsP_6_. This was further confirmed by the observation of both [M-HP^18^O_2_O-H]^−^ and [M-HPO_3_-H]^−^ product ions from ^18^O_10_-labeled 5-InsP_7_, in which two ^18^O atoms were incorporated into each phosphate monoester but no label was present in the diphosphate group (Figure 3A). The HPO_3_ loss intensity increased with collision energy, reaching a maximum at 10 eV, and then decreased again (Figure S5). Figure 3B shows the relative abundances of these HPO_3_ loss. The ratio of [M-HP^18^O_2_O-H]^−^ to [M-HPO_3_-H]^−^ is approximately 1. Specifically, [M-HPO_3_-H]^−^ is slightly more abundant than [M-HP^18^O_2_O-H]^−^ for ^18^O_2_-labeled 5-InsP_7_ (labeling on the β-phosphate of the diphosphate group), and [M-HP^18^O_2_O-H]^−^ is slightly more abundant than [M-HPO_3_-H]^−^ for ^18^O_10_-labeled 5-InsP_7_ (labeling on the monophosphate ester). These results indicate that although five monophosphate esters could theoretically contribute to HPO_3_ loss compared with only one diphosphate, the diphosphate is more labile, resulting in a near 1:1 ratio of product ion abundances rather than 5:1.

**Figure 3:**
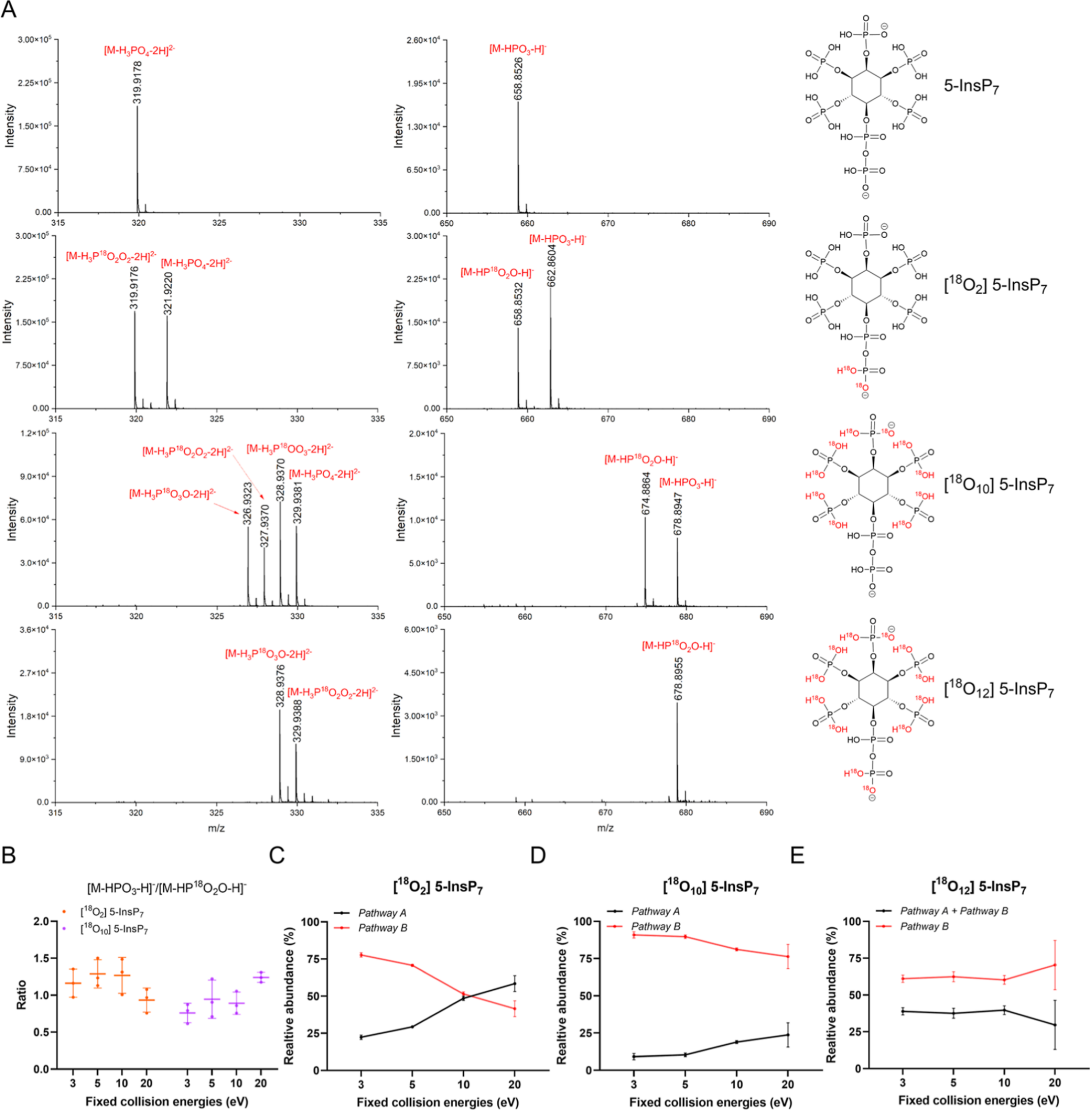
(A) MS/MS fragmentation from the doubly charged precursor of 5-InsP_7_ (at *m*/*z* 368.9066), ^18^O_2_-labeled 5-InsP_7_ (at *m*/*z* 370.9108), ^18^O_10_-labeled 5-InsP_7_ (at *m*/*z* 378.9278), and ^18^O_12_-labeled 5-InsP_7_ (at *m*/*z* 380.9320) using source fragmentation with 10 eV. (B) Ratio of the abundance of [M-HPO_3_-H]^−^ and [M-HP^18^O_2_O-H]^−^ in ^18^O_2_-labeled 5-InsP_7_ and ^18^O_10_-labeled 5-InsP_7_. (C) Relative abundances of the product ions generated from pathways A and B at different collision energies in the ^18^O_2_-labeled 5-InsP_7_. (D) Relative abundances of the product ions generated from pathways A and B at different collision energies in the ^18^O_10_-labeled 5-InsP_7_. (E) Relative abundances of the product ions generated from pathways A and B at different collision energies in the ^18^O_12_-labeled 5-InsP_7_.

The dominant product ion, the doubly charged [M-H_3_PO_4_-2H]^2–^, exhibited more complex fragmentation behavior when produced from ^18^O-labeled 5-InsP_7_ compared to its unlabeled 5-InsP_7_. Previously, we reported the occurrence of “^18^O scrambling” in the ^18^O_2_-labeled 5-InsP_7_, where the ^18^O_2_ labels were incorporated into the terminal phosphate of the diphosphate group.^5^ Notably, related scrambling has been previously observed in ATP and GTP pointing toward a general phenomenon that must be considered in ^18^O labeling studies.^5,43^ To further investigate this phenomenon, we studied the MS2 fragmentation patterns of unlabeled 5-InsP_7_ and compared it to three ^18^O_*x*_-labeled 5-InsP_7s_ (*x* = 2, only β-phosphate labeled; *x* = 10, all phosphates except diphosphate labeled; x = 12, all phosphates, except α-phosphate labeled) and proposed two potential pathways (Scheme 3), helping to rationalize the observed ^18^O redistribution. Synthetic procedures of the ^18^O_*x*_-labeled 5-InsP_7_ (*x* = 10, 12) are described in the Supporting Information. ^18^O_2_-labeled 5-InsP_7_ was synthesized based on a previous publication.^33^ Similar to the case of ^18^O_6_-labeled InsP_3_, pathway A involves elimination or cyclization of adjacent phosphates, leading to the loss of an H_3_PO_4_ group from a monophosphate ester. Depending on whether 5-InsP_7_ carries ^18^O labels on the monophosphate ester, this loss removes either 0 or 2 ^18^O labels. Pathway B involves the neutral loss of HP^18^O_*x*_O_3–*x*_ (*x* = 0 or 2, depending on the location of the ^18^O labels), followed by the subsequent loss of H_2_^18^O or H_2_O via cyclophosphate formation.

**Scheme 3:**
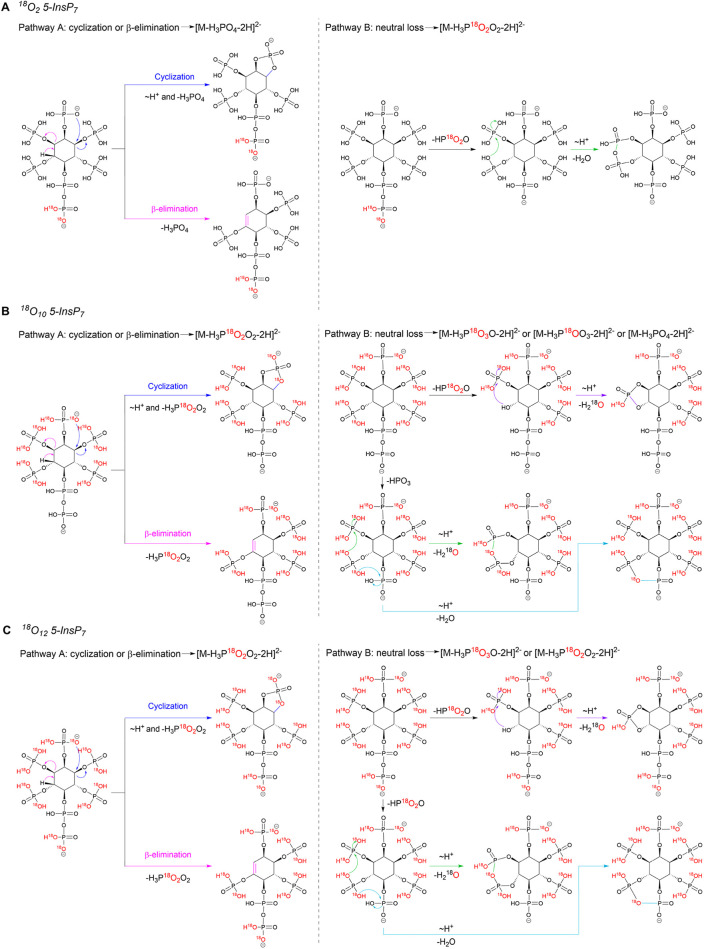
Potential Fragmentation Pathways A and B for ^18^O-Labelled 5-InsP_7_; (A) Fragmentation Pathways Yielding [M-H_3_PO_4_-2H]^2–^ and [M-H_3_P^18^O_2_O_2_-2H]^2–^ from the Precursor Ion of ^18^O_2_-Labelled 5-InsP_7_; (B) Fragmentation Pathways Yielding [M-H_3_PO_4_-2H]^2–^, [M-H_3_P^18^OO_3_-2H]^2–^, [M-H_3_P^18^O_2_O_2_-2H]^2–^, and [M-H_3_P^18^O_3_O-2H]^2–^ from the Precursor Ion of ^18^O_10_-Labelled 5-InsP_7_; (C) Fragmentation Pathways Yielding [M-H_3_P^18^O_2_O_2_-2H]^2–^ and [M-H_3_P^18^O_3_O-2H]^2–^ from the Precursor Ion of ^18^O_12_-Labelled 5-InsP_7_; ∼H^+^ Indicates a Proton Transfer; +H^+^ Indicates the Gain of a Proton; The Ion Structures Shown Represent Only One Possible Isomer, as the Negative Charge Could Also be Located at Other Positions

As shown in Scheme 3, for ^18^O_2_-labeled 5-InsP_7_ (only β-phosphate labeled), pathway A generates the product ion [M-H_3_PO_4_-2H]^2–^ via elimination or cyclization of an adjacent unlabeled monophosphate ester. In pathway B, the neutral loss occurs on the pyrophosphate group, resulting in removal of the 2 ^18^O labels and formation of [M-HP^18^O_2_O-2H]^2–^. This charged intermediate then undergoes an additional H_2_O loss, ultimately yielding [M-H_3_P^18^O_2_O_2_-2H]^2–^. As shown in Figure 3C, pathway B dominates at low collision energies (3–10 eV), while pathway A becomes slightly favored at higher collision energies (up to 20 eV). For ^18^O_10_-labeled 5-InsP_7_ (all phosphates except diphosphate labeled), pathway A generates the product ion [M-H_3_P^18^O_2_O_2_-2H]^2–^ via removal of a labeled monophosphate ester. In pathway B, the labeled monophosphate ester undergoes neutral loss of HP^18^O_2_O, followed by loss of H_2_^18^O from an adjacent labeled monophosphate ester. This yields the compound [M-H_3_P^18^O_3_O-2H]^2–^. Alternatively, the first neutral loss step could occur on the unlabeled diphosphate (loss of HPO_3_), followed by removal of H_2_^18^O from a labeled monophosphate ester or loss of H_2_O from the unlabeled α-phosphate, generating either [M-H_3_P^18^OO_3_-2H]^2–^ or [M-H_3_PO_4_-2H]^2–^. As shown in Figure 3D, pathway B is dominant. A similar mechanism is observed for ^18^O_12_-labeled 5-InsP_7_ (all phosphates, except α-phosphate labeled), producing two expected product ions [M-H_3_P^18^O_2_O_2_-2H]^2–^ and [M-H_3_P^18^O_3_O-2H]^2–^. Product ion [M-H_3_P^18^O_2_O_2_-2H]^2–^ can arise from either pathway A (via loss of H_3_P^18^O_2_O_2_ from an adjacent labeled monophosphate ester) or pathway B (via loss of HP^18^O_2_O from the labeled β-phosphate followed by H_2_O loss from the unlabeled α-phosphate). In contrast, [M-H_3_P^18^O_3_O-2H]^2–^ is generated exclusively through pathway B, either via sequential loss of HP^18^O_2_O from the labeled β-phosphate and H_2_^18^O loss from an adjacent labeled monophosphate ester, or initial loss of HP^18^O_2_O from a labeled monophosphate ester followed by loss of H_2_^18^O from an adjacent labeled monophosphate ester. Again, pathway B is clearly dominant (Figure 3E).

In summary, two main pathways are proposed: (A) cyclization or β-elimination involving adjacent monophosphate esters and (B) neutral loss of HP^18^O_*x*_O_3‑x_ (x = 0 or 2) followed by further loss of H_2_^18^O or H_2_O. These pathways explain the observed redistribution of ^18^O labels during ESI MS/MS fragmentation, though it is not possible currently to confidently assign the exact mechanism. Moreover, the structural representations in the schemes depict only one possible isomeric form, whereas the actual fragmentation could occur at multiple equivalent sites (e.g., H_3_P^18^O_2_O_2_ loss by β-elimination in the ^18^O_12_-labeled InsP_6_ could occur at any adjacent pair of monophosphate esters), however always producing ions of the same mass. Comparison of the relative abundances of these fragment ions allows estimation of the competing fragmentation pathways. Pathway B dominates in ^18^O-labeled InsP_3_, InsP_6_, and 5-InsP_7_, with the exception that pathway A becomes slightly favored in ^18^O_2_-labeled 5-InsP_7_ at higher collision energies (20 eV).

Importantly, these insights into fragmentation behavior and label redistribution improve our ability to predict diagnostic fragment ions. Previous publications optimized [M-H_3_PO_4_-H]^−^ as the product ion for MRM analysis of InsP_3_, [M-HPO_3_-H_3_PO_4_-H]^−^ for InsP_6_, and [M-H_3_PO_4_-2H]^2–^ for InsP_7_.^6,13^ However, our fragmentation mechanism study here indicates that potential ^18^O redistribution must be taken into account to achieve accurate quantitative analysis when these product ions are selected in the MRM methods, as demonstrated in our earlier study.^5^ Understanding the fragmentation mechanism provides a rational basis for selecting robust MRM transitions, thereby enhancing the reliability and accuracy of quantitative analyses of inositol phosphates. It also guides the selection of suitable product ions for quantitation when synthetic ^18^O-labeled InsPs and PP-InsPs are used as internal standards. This aspect will be addressed in the following section. Furthermore, we investigated the potential hydrolytic back-exchange of ^18^O with ^16^O (Table S2). No ^18^O/^16^O exchange was observed in aqueous stock solutions over 11 months for ^18^O_6_-labeled InsP_3_ and ^18^O_2_-labeled 5-InsP_7,_ 8 months for ^18^O_12_-labeled InsP_6_, and 2 months for ^18^O_10_-labeled 5-InsP_7_ and ^18^O_12_-labeled 5-InsP_7_. The different time courses reflect the time since synthesis rather than exchange of the ^18^O labels.

### 3.2 Method Optimization of ^18^O-Labeled Standards for Quantitative Analysis of InsPs and PP-InsPs by CE-QqQ

As discussed in the introduction, ^18^O-labeling of InsP and PP-InsP standards could be an effective alternative to ^13^C-labeled analogs for quantitative analysis due to their lower cost and enzyme-free synthesis approach. However, the redistribution of ^18^O within dominant MS/MS product ions (the loss of an H_3_PO_4_ group or subsequent loss of HPO_3_ and H_3_PO_4_ in InsP_6_) is a challenge for their application in quantitative CE-MS/MS analysis. Achieving the highest sensitivity typically requires the use of a CE-QqQ system, which relies on such precursor-product ion transitions. Thus, identifying the optimal transition without interference or a loss of specificity is important.

Based on the fragmentation patterns of ^18^O_6_-labeled InsP_3_, ^18^O_12_-labeled InsP_6_, and ^18^O_12_-labeled InsP_7_ described above, the loss of the HP^18^O_2_O group was identified as a promising candidate for monitoring. For example, ^18^O_12_-labeled 5-InsP_7_, where two ^18^O labels were introduced into all the phosphate groups attached to the inositol ring and the terminal phosphate of the diphosphate group (thus excluding the α-phosphate), yielded a single transition corresponding to [M-HP^18^O_2_O-H]^−^. In contrast, both [M-HP^18^O_2_O-H]^−^ and [M-HPO_3_-H]^−^ were observed for ^18^O_2_-labeled 5-InsP_7_ (^18^O labeling limited to the terminal pyrophosphate) and ^18^O_10_-labeled 5-InsP_7_ (^18^O labeling limited to phosphate esters attached to inositol) (Figure 3A). These findings suggest that ^18^O labeling must include both the inositol-bound phosphates and the terminal phosphate of the pyrophosphate group to ensure accurate quantitative analysis, in which case the HP^18^O_2_O loss can serve as a reliable product ion. After the precursor and product ions were defined, the collision energy (1–60 eV) and cell accelerator voltage (1–8 V) were optimized using Agilent Optimizer to maximize product ion (loss of HP^18^O_2_O) intensity. The optimization was performed with 200 μM solutions of ^18^O_6_-labeled Ins­(1,4,5)­P_3_, ^18^O_12_-labeled InsP_6_, and ^18^O_12_-labeled 5-InsP_7_, respectively. The optimized parameters for the loss of [M-HP^18^O_2_O-H]^−^ of ^18^O-labeled standards are summarized in Table S3. Representative extracted ion electropherograms (EIEs) of ^18^O_6_-labeled Ins­(1,4,5) P_3_, ^18^O_12_-labeled InsP_6_, and ^18^O_12_-labeled 5-InsP_7_ are shown in Figure 4.

**Figure 4:**
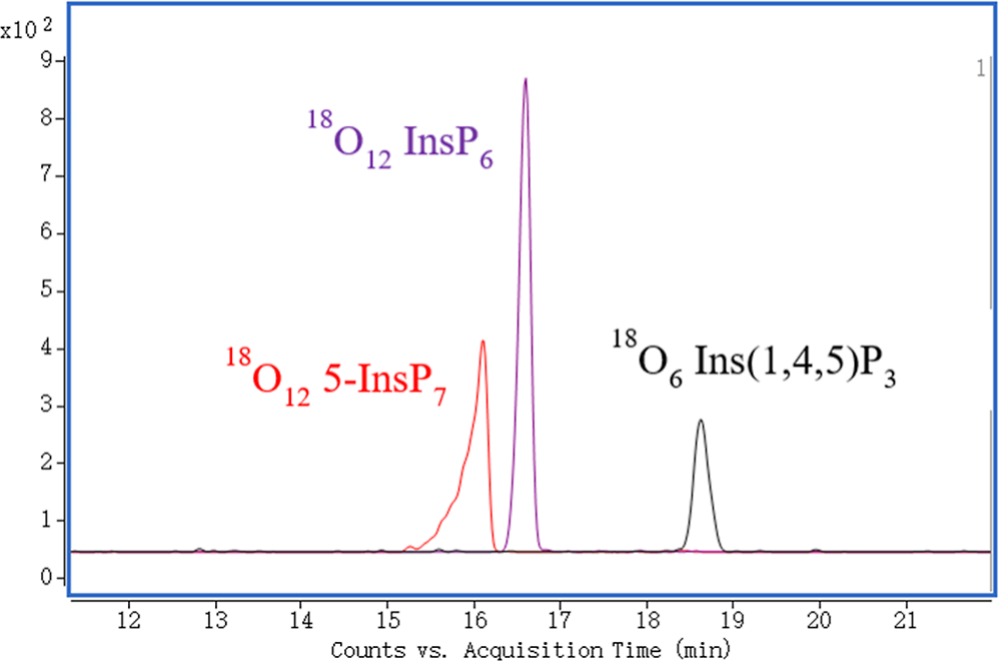
Extracted ion electropherograms (EIEs) of ^18^O_6_-labeled Ins­(1,4,5)­P_3_, ^18^O_12_-labeled InsP_6_, and ^18^O-labeled 5-InsP_7_ at a concentration of 5 μM.

Previously, the most abundant product ions were used for quantitative analysis of InsPs and PP-InsPs with ^13^C_6_-labeled internal standards: [M-HPO_3_-H_3_PO_4_-H]^−^ for InsP_6_ and ^13^C_6_-labeled InsP_6_, and [M-H_3_PO_4_-2H]^2–^ for InsP_7_ and ^13^C_6_-labeled InsP_7_. To minimize the influence of the ^18^O redistribution on quantitation, we selected [M-HPO_3_-H]^−^ as the product ion (corresponding to [M-HP^18^O_2_O-H]^−^ in the ^18^O-labeled reference) and optimized the MRM parameter to maximize ion intensity as discussed above. To evaluate the sensitivity trade-off between this alternative transition and previously established ones,^6,13^ we compared the abundances of the two product ions using ^13^C_6_-labeled standards. ^18^O-labeled standards could not be used for this evaluation as ^18^O redistribution would confound the comparison. Both product ions were monitored under optimized MRM conditions (Table S4) at three concentration levels (1, 5, and 20 μM) of the ^13^C_6_-labeled standards. Since a ^13^C-labeled Ins­(1,4,5)­P_3_ standard was not available at the time,^30^ the unlabeled Ins­(1,4,5)­P_3_ was used instead. All standard concentrations were determined by quantitative nuclear magnetic resonance spectroscopy (qNMR), and measurements were performed with CE-QqQ in six replicates per concentration.

As shown in Figure 5, the peak area ratios between the two product ions were reproducible across replicates, concentrations, and measurement days. The relative standard deviations (RSDs) of these ratios are summarized in Table 3. The intraday precision (*n* = 6 replicates) ranged from 2.3 to 10.9%, the interday precision (*n* = 3 days, each with six replicates) ranged from 2.3 and 17.7%, and cross-concentration precision ranged from 3.7 to 14.4%. For InsP_7_, the average abundance ratio of [M-H_3_PO_4_-2H]^2–^ (previously optimized) and [M-HPO_3_-H]^−^ (this study) was 17.2 (*n* = 54 replicates), indicating that the alternative transition is ∼17-fold less intense. By contrast, for InsP_6_ and InsP_3_, the sensitivity was similar, with average ratios of 0.9 and 1.2 (*n* = 54 replicates), respectively. These results support the use of [M-HPO_3_-H]^−^ as a reliable alternative product ion for quantitative analysis of InsPs and PP-InsPs, with the advantage of avoiding ^18^O redistribution while maintaining analytical robustness.

**Figure 5:**
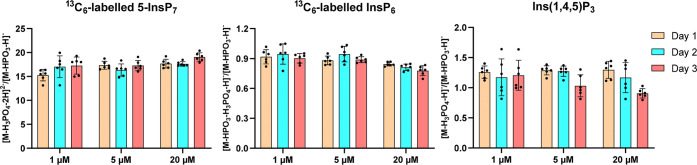
Intra- and inter-day repeatability of the ratio between two product ions analyzed by CE-QqQ. Data are presented as means ± SD (intraday *n* = 3, interday *n* = 6).

**Table 3:** Relative Standard Deviations of Peak Area Ratios between Two Product Ions and Average Ratios for Each InsPs and PP-InsPs

	concentration (μM)	^13^C_6_ 5-InsP_7_	^13^C_6_ InsP_6_	Ins(1,4,5)P_3_
		[M-H_3_PO_4_-2H]^2–^/[M-HPO_3_-H]^−^	[M-HPO_3_-H_3_PO_4_-H]^−^/[M-HPO_3_-H]^−^	[M-H_3_PO_4_-H]^−^/[M-HPO_3_-H]^−^
intraday RSD (*n* = 6) (%)	1	7.8	7.8	8.1
	5	4.8	4.9	5.9
	20	5.2	2.3	10.9
interday (RSD) (*n* = 3, each with six replicates) (%)	1	6.6	2.3	3.5
	5	3.2	3.8	12.0
	20	4.5	4.1	17.7
intraday cross-concentration (RSD) (1 μM, 5 μM and 20 μM, each concentration with six replicates) (%)	1, 5, 20	3.7	4.1	14.4
average of the ratio (*n* = 54)		17.2	0.9	1.2

### 3.3 Comparative Quantitation of InsPs and PP-InsPs Using ^18^O- and ^13^C-Labeled Standards in Complex Matrices

To evaluate the utility of the ^18^O-labeled standards and the new MS method for quantifying InsP_6_ and InsP_7_ in biological extracts, we spiked both ^18^O-labeled standard and ^13^C-labeled standards into *Saccharomyces cerevisiae*, HCT116 cells, and *Arabidopsis thaliana* samples to quantify InsP and PP-InsP abundances. *S. cerevisiae* and HCT116 cells samples were obtained from Mayer’s lab, as previously described.^5^ *A. thaliana* samples were obtained from Laha’s lab, as previously described.^29^ In CE-QqQ analysis, the MRM transitions for InsPs were identical to those reported previously (Table S5).^6,13^ Keeping the MRM transitions unchanged preserves the detection sensitivity and provides comparable limits of detection and quantitation. The representative extracted ion electropherograms (EIEs) are shown in Figure S6. For quantitation, ^18^O-labeled standards were used as internal references instead of previously used ^13^C-labeled references, and the optimized MRM transitions for ^18^O-labeled references were applied. Normalization factors for the optimized MRM transitions derived from the abundance ratio (Table 3) were applied. Specifically, for ^18^O_12_-labeled 5-InsP_7_, we spiked in a 17.2-fold higher concentration of ^13^C_6_-labeled 5-InsP_7_ but quantified concentrations using the concentration value of ^13^C_6_-labeled 5-InsP_7_. Therefore, the ^18^O reference peak area was normalized using a factor of 17.2. For InsP_6_, a 0.9-fold adjustment was applied analogously. As shown in Figure 6, quantitation of InsP_6_ using the ^18^O reference produced results consistent with those obtained using the ^13^C reference across all the experimental biological samples. In contrast, InsP_7_ quantitation based on the ^18^O reference yielded values approximately 3-fold higher than those obtained by using the ^13^C reference. The explanation is that the stored ^13^C_6_-labeled 5-InsP_7_ had undergone partial hydrolysis of the disphosphate, as evidenced by the presence of a ^13^C-labeled InsP_6_ with a peak area corresponding to roughly 1/3 of that of ^13^C-labeled 5-InsP_7_.

**Figure 6:**
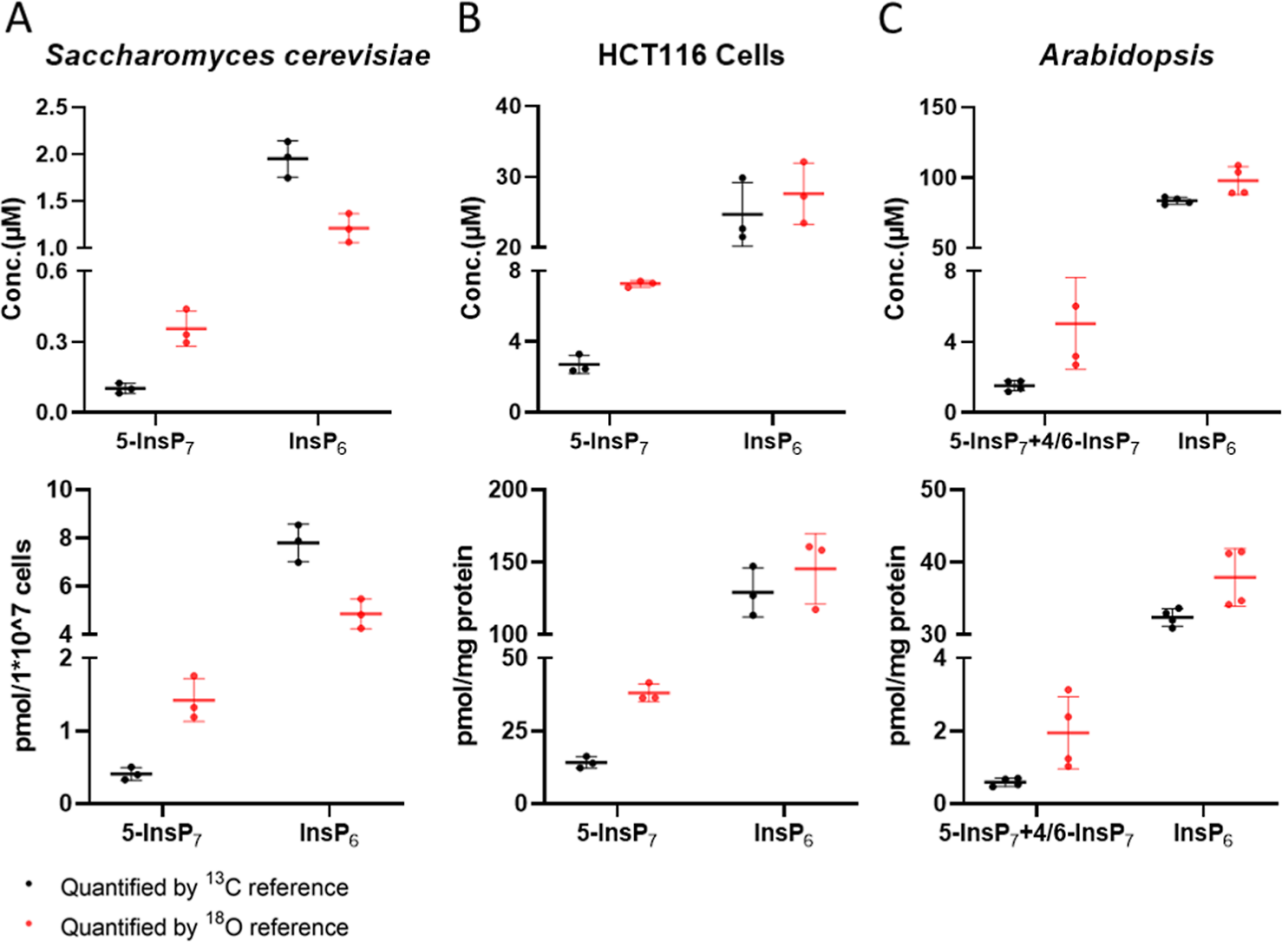
Comparative quantitation of InsP_7_ and InsP_6_ using ^18^O- and ^13^C-labeled standards in *Saccharomyces cerevisiae* (A, *n* = 3), HCT116 cells (*n* = 3), and *Arabidopsis thaliana* (*n* = 4). In *Saccharomyces cerevisiae* and HCT116 cells, 5-InsP_7_ is the major InsP_7_ isomer. In *Arabidopsis thaliana*, 4/6-InsP_7_ is present in comparable amounts as previously reported. The assignment of isomers was performed by using synthetic isotope labeled internal standards.

## 4 Conclusions

This study provides a comprehensive evaluation of ^18^O-labeled InsPs and PP-InsPs as internal standards for quantitative CE-MS analysis. By synthesizing strategically labeled standards and comparing their MS2 fragmentation behavior, we elucidated key fragmentation pathways responsible for the ^18^O redistribution, including charge-driven dissociation and sequential losses of HPO_3_ and H_2_O groups. This study unveils complex fragmentation patterns that cannot be seen with unlabeled molecules. These mechanistic insights guided the identification of specific transitions, such as the loss of HPO_3_, to minimize isotope scrambling and improve analytical reliability. Furthermore, by establishing normalization factors based on the consistent response ratios between ^18^O- and ^13^C-labeled standards, we demonstrate that ^18^O-labeled standards can serve as a cost-effective and practical alternative for the accurate quantitation of InsPs and PP-InsPs in biological samples.

## Supplementary Material





## Notes

Theoretical and observed abundant precursor *m*/*z* values of InsPs and PP-InsPs; stability of ^18^O-labeled InsPs and PP-InsPs; MRM transitions settings of InsPs and PP-InsPs; collision-cell fragmentation experiments with InsPs and PP-InsPs; extracted ion electropherograms of InsP_7_ and InsP_6_ from biological extracts; source of chemicals; synthetic procedures for ^18^O-labeled InsPs and PP-InsPs; and NMR spectra (PDF)
Source data used to make the figures (XLSX)
