# Supplementary material for: Establishing 18O‑Labeled Inositol Phosphates for Quantitative Capillary Electrophoresis-Mass Spectrometry: Fragmentation Pathways and Comparison with 13C‑Labeled Analogs
Source: Anal Chem. 2025 Nov 5;97(45):25282–94. doi: 10.1021/acs.analchem.5c05114 (PMC12631729; doi:10.1021/acs.analchem.5c05114)
Supplement: Supplementary file 1 [file ac5c05114_si_001.pdf]

Supplementary Information

Establishing ^{18}O-labelled Inositol Phosphates for Quantitative Capillary Electrophoresis-Mass Spectrometry: Fragmentation Pathways and Comparison with ^{13}C-labelled Analogs

Guizhen Liu[1,2], Tobias Dürr-Mayer[1,2], Mengsi Lu[1], Henning J. Jessen[1,2]*

[1] Institute of Organic Chemistry, University of Freiburg, Albertstrasse 21, 79104 Freiburg, Germany

[2]CIBSS, Centre for Integrative Biological Signalling Studies, University of Freiburg, 79104 Freiburg, Germany

Contents

1. Precursor *m/z* values of InsPs and PP-InsPs S3

2. Stability of ^{18}O-labelled InsPs and PP-InsPs S3

3. MRM transitions settings of InsPs and PP-InsPs.. S4

4. Collision-cell fragmentation experiments with InsPs and PP-InsPs.................................... S6

5. Extracted ion electropherograms of (^{18}O-labelled) $InsP_7$ and $InsP_6$ from biological extracts S10

6. Synthesis of ^{18}O-labelled InsPs and PP-InsPs S11

7. NMR-spectra S17

1. Precursor *m/z* values of InsPs and PP-InsPs

Supplementary Table S1. The theoretical and observed abundant precursor *m/z* values of InsPs and PP-InsPs by CE-QTOF

Molecular name	Charge state	Theoretical *m/z*	Observed mass *m/z*
Ins(1,4,5)P_3	1	418.9551	418.9550
$^{18}O_6$ Ins(1,4,5)P_3	1	430.9806	430.9804
$InsP_6$	2	328.9234	328.9233
$^{18}O_{12}$ $InsP_6$	2	340.9489	340.9484
5-$InsP_7$	2	368.9066	368.9061
$^{18}O_2$ 5-$InsP_7$	2	370.9108	370.9105
$^{18}O_{10}$ 5-$InsP_7$	2	378.9278	378.9271
$^{18}O_{12}$ 5-$InsP_7$	2	380.9320	380.9320

2. Stability of ^{18}O-labelled InsPs and PP-InsPs

Supplementary Table S2. Stability of ^{18}O-labelled InsPs and PP-InsPs

	**$^{18}O_6$ Ins(1,4,5)P_3**	
	M+12 (due to 6 ^{18}O labels)	M+10
t=0	92.5%	6.5%
t=11 months	94.3%	5.7%
	**$^{18}O_{12}$ $InsP_6$**	
	M+24 (due to 6 ^{18}O labels)	M+22
t=0	92.0%	8.0%
t=8 months	91.1%	8.9%
	**$^{18}O_2$ 5-$InsP_7$**	
	M+4 (due to 2 ^{18}O labels)	M+2
t=0	98.4%	1.6%
t=11 months	97.9%	2.1%
	**$^{18}O_{10}$ 5-$InsP_7$**	
	M+20 (due to 10 ^{18}O labels)	M+18
t=0	85.8%	14.2%
t=2 months	86.3%	13.7%
	**$^{18}O_{12}$ 5-$InsP_7$**	
	M+24 (due to 12 ^{18}O labels)	M+22
t=0	68.0%	32%
t=2 months	68.2%	31.8%

3. MRM transitions settings of InsPs and PP-InsPs

Supplementary Table S3. Optimized MRM transitions setting of ^{18}O-labelled InsPs and PP-InsPs

Molecular name	Precursor ion	Product ion	Type of transition	Collision energy (V)	Cell accelerator voltage (V)
$^{18}O_6$-labelled $Ins(1,4,5)P_3$	430.9	347.4	$[M\text{-}H]^- \rightarrow [M\text{-}HP^{18}O_2O\text{-}H]^-$	26	1
$^{18}O_{12}$-labelled $InsP_6$	340.9	599.3	$[M\text{-}2H]^{2-} \rightarrow [M\text{-}HP^{18}O_2O\text{-}H]^-$	11	2
$^{18}O_{12}$-labelled 5-$InsP_7$	380.9	678.9	$[M\text{-}2H]^{2-} \rightarrow [M\text{-}HP^{18}O_2O\text{-}H]^-$	12	4

Note: Frag 166V; Precursor ion MS1 and product ion resolutions are unit.

Supplementary Table S4. MRM transitions setting of InsPs and PP-InsPs for two different product ions

Molecular name	Precursor ion	Product ion	Type of transition	Collision energy (V)	Cell accelerator voltage (V)	Notes
$Ins(1,4,5)P_3$	418.9	320.8	$[M\text{-}H]^- \rightarrow [M\text{-}H_3PO_4\text{-}H]^-$	17	4	Optimized previously
$Ins(1,4,5)P_3$	418.9	338.9	$[M\text{-}H]^- \rightarrow [M\text{-}HPO_3\text{-}H]^-$	26	1	this study
$^{13}C_6$-labelled $InsP_6$	331.9	486.9	$[M\text{-}2H]^{2-} \rightarrow [M\text{-}HPO_3\text{-}H_3PO_4\text{-}H]^-$	13	4	Optimized previously
$^{13}C_6$-labelled $InsP_6$	331.9	585.3	$[M\text{-}2H]^{2-} \rightarrow [M\text{-}HPO_3\text{-}H]^-$	11	2	this study
$^{13}C_6$-labelled 5-$InsP_7$	371.9	322.9	$[M\text{-}2H]^{2-} \rightarrow [M\text{-}H_3PO_4\text{-}2H]^{2-}$	10	3	Optimized previously
$^{13}C_6$-labelled 5-$InsP_7$	371.9	664.9	$[M\text{-}2H]^{2-} \rightarrow [M\text{-}HPO_3\text{-}H]^-$	12	4	this study

Note: Frag 166V; Precursor ion MS1 and product ion resolutions are unit.

Supplementary Table S5. MRM transitions setting of InsPs and PP-InsPs for quantitative analysis using ^{18}O-labelled reference and ^{13}C-labelled reference for biological samples

Molecular name	Precursor ion	Product ion	Type of transition	Collision energy (V)	Cell accelerator voltage (V)
$^{13}C_6$-labelled $InsP_6$	331.9	486.9	$[M\text{-}2H]^{2-} \rightarrow [M\text{-}HPO_3\text{-}H_3PO_4\text{-}H]^{-}$	13	4
$^{18}O_{12}$-labelled $InsP_6$	340.9	599.3	$[M\text{-}2H]^{2-} \rightarrow [M\text{-}HP^{18}O_2O\text{-}H]^{-}$	11	2
$InsP_6$	328.9	480.9	$[M\text{-}2H]^{2-} \rightarrow [M\text{-}HPO_3\text{-}H_3PO_4\text{-}H]^{-}$	13	4
$^{13}C_6$-labelled 5-$InsP_7$	371.9	322.9	$[M\text{-}2H]^{2-} \rightarrow [M\text{-}H_3PO_4\text{-}2H]^{2-}$	10	3
$^{18}O_{12}$-labelled 5-$InsP_7$	380.9	678.9	$[M\text{-}2H]^{2-} \rightarrow [M\text{-}HP^{18}O_2O\text{-}H]^{-}$	12	4
$InsP_7$	368.9	319.9	$[M\text{-}2H]^{2-} \rightarrow [M\text{-}H_3PO_4\text{-}2H]^{2-}$	10	3

Note: Frag 166V; Precursor ion MS1 and product ion resolutions are unit.

4. Collision-cell fragmentation experiments with InsPs and PP-InsPs

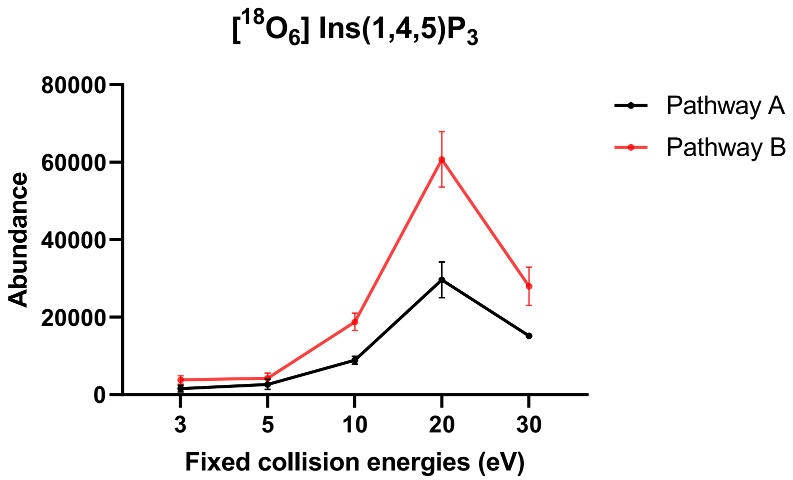

Supplementary Figure S1. In collision-cell energies experiments, the abundance of both product ions varied with collision energy.

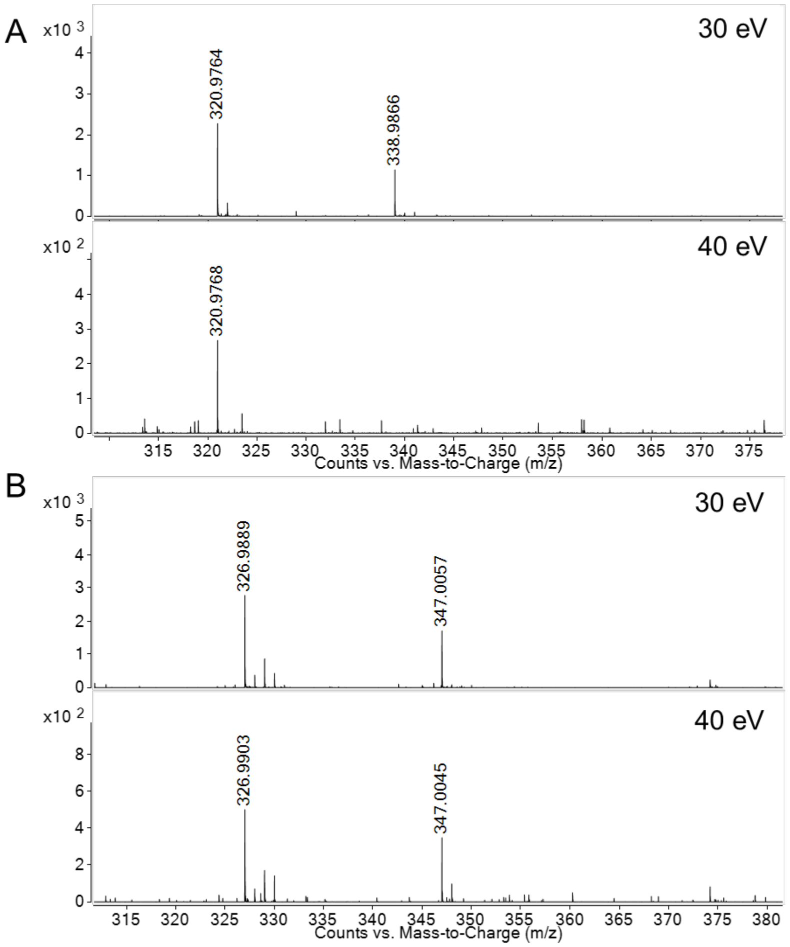

Supplementary Figure S2. The MS/MS spectra of the singly charged precursor of Ins(1,4,5)P_3 (A) and $^{18}O_6$-labelled Ins(1,4,5)P_3 (B) acquired at collision energies of 30 eV and 40 eV.

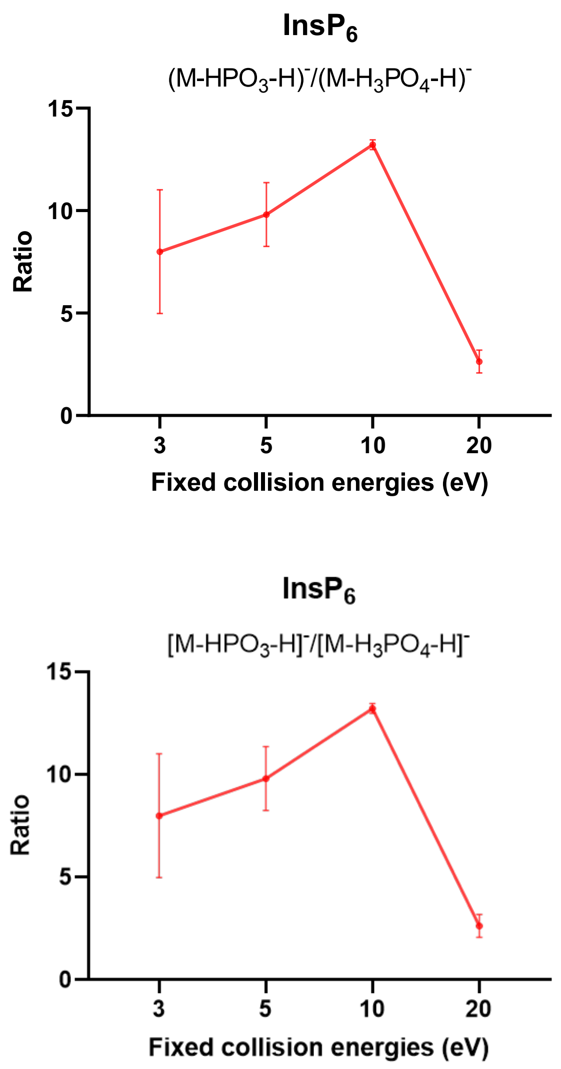

Supplementary Figure S3. Ratio of the abundance of two product ions $[M\text{-}HPO_3\text{-}H]^-$ and $[M\text{-}H_3PO_4\text{-}H]^-$ at different collision energies in unlabelled $InsP_6$.

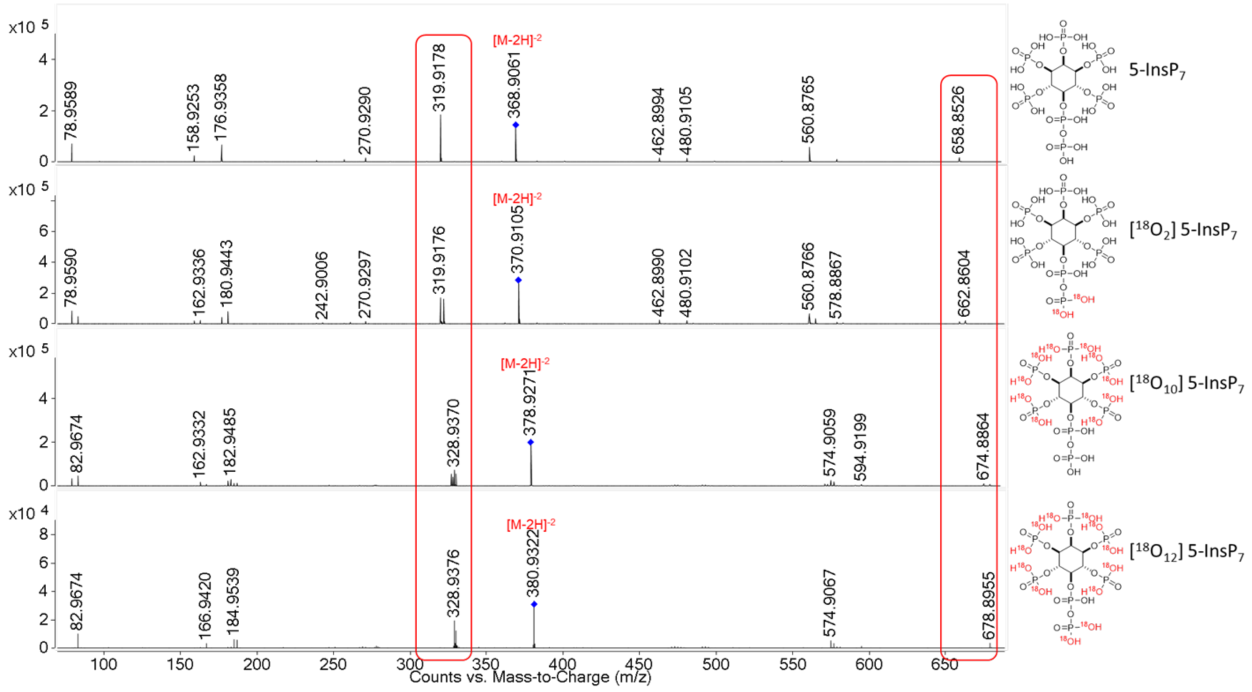

Supplementary Figure S4. The MS/MS spectra of doubly charged precursor of 5-InsP$_7$ and ^{18}O-labelled 5-InsP$_7$ obtained using source fragmentation at 10 eV. The zoom of the two regions marked in red are shown in Figure 3A.

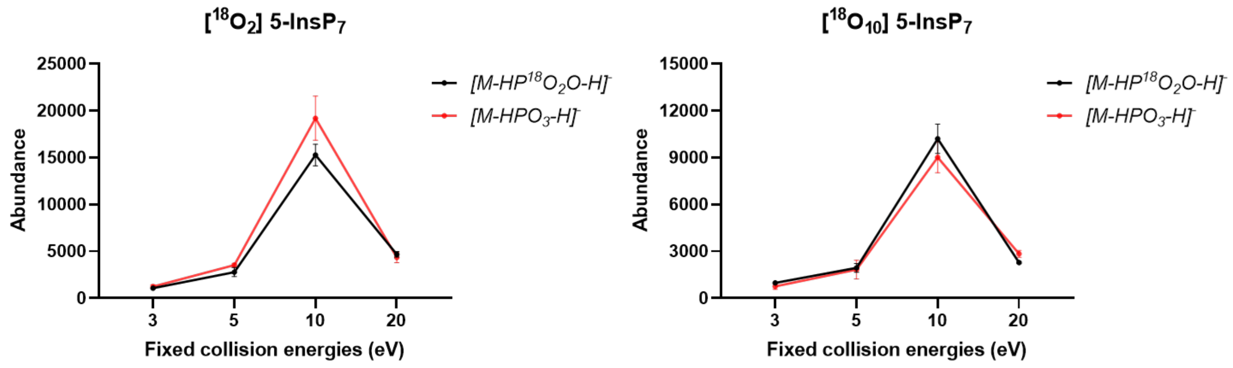

Supplementary Figure S5. Abundances of [M-HPO$_3$-H]$^-$ and [M-HP^{18}O$_2$O-H]$^-$ at different collision energies.

5. Extracted ion electropherograms of (^{18}O-labelled) $InsP_7$ and $InsP_6$ from biological extracts

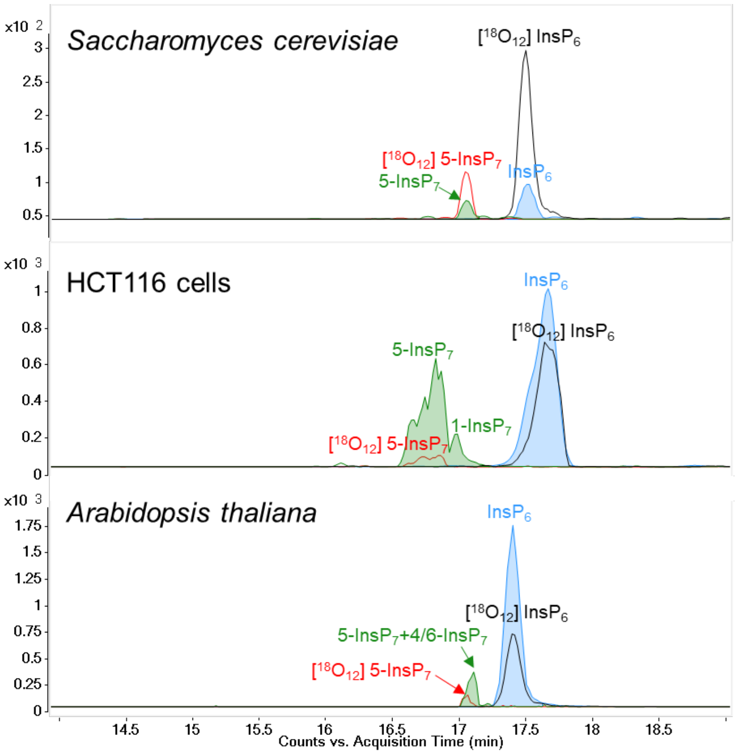

Supplementary Figure S6. Extracted ion electropherograms (EIEs) of $InsP_7$ (green trace) and $InsP_6$ (blue trace) in *Saccharomyces cerevisiae*, HCT116, and *Arabidopsis thaliana* using ^{18}O-labelled references.

6. Synthesis of ^{18}O-labelled InsPs and PP-InsPs

Supplementary Table S6. Source of Chemicals

Chemicals	Supplier	Country
DBU	Sigma	Germany
BSTFA	Fisher Scientific	Germany
ETT	ChemPUR	Germany
TFA	Fisher Scientific	Germany
*m*CPBA	Sigma	Germany
$NaHCO_3$	Fisher Scientific	Belgium
Pd/C (10 wt.% loading)	Thermo Fisher Scientific	United Kingdom
TEAA buffer pH 7.0 (1 M)	AppliChem	Germany
MeCN	Thermo Scientific	Germany
MeOH	Fisher Scientific	Germany
*t*BuOH	Carl Roth	Germany
CH_2Cl_2	Fisher Scientific	Germany
2,4,6-PMB_3-5-Fm_2-$InsP_1$	Synthesized[1]	Germany
$^{18}O_2$-dibenzyl-*N,N*-diisopropyl phosphoramidite	Synthesized[2]	Germany
AB_2-*N,N*-diisopropyl phosphoramidite	Synthesized[3]	Germany

[1] S. Capolicchio, H. Wang, D. T. Thakor, S. B. Shears, H. J. Jessen, *Angew. Chem. Int. Ed.* **2014**, *53*, 9508.

[2] A. Hofer, G. S. Cremosnik, A. C. Müller, R. Giambruno, C. Trefzer, G. Superti-Furga, K. L. Bennett, H. J. Jessen, *Chem. Eur. J.* **2015**, *21*, 10116.

[3] H. J. Jessen, T. Schulz, J. Balzarini, C. Meier, *Angew. Chem. Int. Ed.* **2008**, *47*, 8719.

5-Fm_2-$InsP_1$

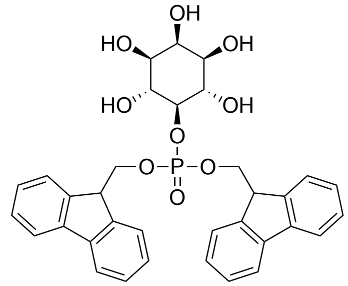

Chemical Formula: $C_{34}H_{33}O_9P$
Exact Mass: 616,1862

2,4,6-PMB_3-5-Fm_2-$InsP_1$ (1.69 g, 1.73 mmol) was dissolved in CH_2Cl_2 (40 ml). TFA (2.1 ml) was added and stirred for 3 h. The solvent was removed *in vacuo.* The residue was suspended with acetone (7 ml) and precipitated using Et_2O (40 ml). The precipitate was collected and washed with Et_2O. Drying *in vacuo* gave the product [1.04 g, 1.69 mmol, 98%] as a white solid.

^{1}H-NMR (400 MHz, DMSO-d_6): δ = 7.86 (m, 4H), 7.59 (m, 4H), 7.39 (m, 4H), 7.28 (m, 4H), 5.01 (d, *J* = 5.6 Hz, 2H), 4.69 (d, *J* = 3.6 Hz, 1H), 4.65 (d, *J* = 5.9 Hz, 2H), 4.29 (m, 4H), 4.20 (t, *J* = 6.7 Hz, 2H), 3.97 (dd, *J* = 9.3, 9.3 Hz, 1H), 3.74 (dd, *J* = 2.9, 2.9 Hz, 1H), 3.62 (ddd, *J* = 9.5, 9.4, 5.5 Hz, 2H), 3.23 ppm (ddd, *J* = 9.7, 5.9, 2.6 Hz, 2H). **^{31}P{^{1}H}-NMR** (162 MHz, DMSO-d_6): δ = -1.93 ppm. **^{31}P-NMR** (162 MHz, DMSO-d_6): δ = -1.93 ppm (dtt, *J* = 6.4, 6.4, 6.3 Hz). **^{13}C-NMR** (101 MHz, DMSO-d_6): δ = 144.0, 144.0, 141.3, 141.2, 128.1, 127.5, 125.8, 125.7, 120.5, 83.7, 83.7, 72.9, 72.0, 71.8, 71.8, 68.7, 68.6, 48.0, 47.9 ppm. **HRMS (ESI):** *m/z* calcd for $C_{34}H_{34}O_9P$ $[M + H^+]^+$: 617.1935, found: 617.1936.

[$^{18}O_{10}$]Bn_{10}-5-Fm_2-$InsP_6$

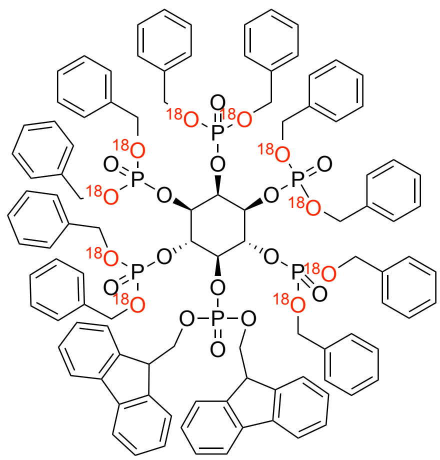

Chemical Formula: $C_{104}H_{98}O_{14}{}^{18}O_{10}P_6$
Exact Mass: 1936,5298

5-Fm_2-$InsP_1$ (154 mg, 250 µmol, 1.0 eq.) and $^{18}O_2$-dibenzyl-*N,N*-diisopropyl phosphoramidite (836 mg, 2.3 mmol, 9.0 eq.) were coevaporated using MeCN (3 x 5 ml). The residue was dissolved/suspended in MeCN (24 ml) and cooled with an ice-bath. A solution of ETT in MeCN (500 mM, 6.8 ml, 439 mg, 3.4 mmol, 13.5 eq.) was added and stirred for 3 h. *m*CPBA (≤ 77%, 504 mg, 2.3 mmol, 9.0 eq.) was slowly added under cooling with an ice-bath and stirred for 10 min. The reaction mixture was subjected to Celite and purified using a PuriFlash Column (15µ C18 AQ, MeCN gradient (40-100%)). The solvent was removed under reduced pressure to give the product [351 mg, 181 µmol, 73%] as a white solid.

^{1}H-NMR (400 MHz, $CDCl_3$): δ = 7.59 (m, 4H), 7.44 (m, 2H), 7.31 (m, 4H), 7.28 – 6.99 (m, 56H), 5.66 (m, 1H), 5.30 – 4.75 (m, 22H), 4.35 (m, 3H), 4.20 (m, 4H), 3.93 ppm (dd, *J* = 6.8, 6.8 Hz, 2H). **^{31}P{^{1}H}-NMR** (162 MHz, $CDCl_3$): δ = -0.86 (s, 2P), -1.47 (s, 1P), -1.56 (s, 2P), -2.50 ppm (s, 1P). **^{31}P-NMR** (162 MHz, $CDCl_3$): δ = -0.86 (dp, *J* = 9.0, 8.6 Hz, 2P), -1.54 (m, 3P), -2.50 ppm (m, 1P). **^{13}C-NMR** (101 MHz, $CDCl_3$): δ = 143.4, 141.3, 136.2 – 135.7 (m), 128.9 – 127.8 (m), 127.6, 127.0, 125.5, 119.8, 75.7, 74.7, 73.6, 70.4 – 69.3 ppm (m). **HRMS (ESI):** *m/z* calcd for $C_{104}H_{99}O_{14}{}^{18}O_{10}NaP_6$ [M + Na^+]$^+$: 1960.5269, found: 1960.5219.

[$^{18}O_{10}$]Bn$_{10}$-5-AB$_2$-InsP$_7$

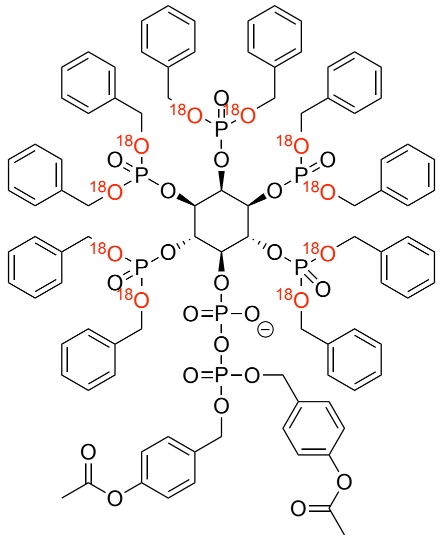

Chemical Formula: $C_{94}H_{94}O_{21}{}^{18}O_{10}P_7^-$
Exact Mass: 1955,4372

[$^{18}O_{10}$]Bn$_{10}$-5-Fm$_2$-InsP$_6$ (189 mg, 94 µmol, 1.0 eq.) was coevaporated using MeCN (3 x 2 ml) and the residue dissolved in MeCN (4.0 ml). DBU (56 µl, 57 mg, 376 µmol, 4.0 eq.) and BSTFA (101 µl, 97 mg, 376 µmol, 4.0 eq.) were added and stirred for 10 min. A mixture of TFA (29 µl, 43 µg, 376 µmol, 4.0 eq.) and MeOH (20 µl) was added and stirred for 1 min. The solvent was removed *in vacuo*. The residue was coevaporated using MeCN (2 x 0.5 ml) and then dissolved in MeCN (2.0 ml). In another flask, AB$_2$-*N,N*-diisopropyl phosphoramidite (87 mg, 188 µmol, 2.0 eq.) was coevaporated using MeCN (3 x 2 ml) and dissolved in MeCN (2.0 ml). This solution was added to the reaction flask. A solution of ETT in MeCN (500 mM, 376 µl, 25 mg, 188 µmol, 2.0 eq.) was added and stirred for 20 min. *m*CPBA (≤ 77%, 42 mg, 188 µmol, 2.0 eq.) was slowly added under cooling with an ice-bath and stirred for 10 min. The reaction mixture was subjected to Celite and purified using a PuriFlash Column (15µ C18 AQ, MeCN gradient (25-100%), 10% TEAA (100 mM, pH 7.0)). The solvent was removed under reduced pressure to give the product [132 mg, 64 µmol, 68%] as a sticky oil.

^{1}H-NMR (400 MHz, CD_3CN): δ = 7.32 – 7.18 (m, 54H), 6.95 (m, 4H), 5.20 – 5.00 (m, 24H), 2.22 ppm (s, 6H). **^{31}P{^{1}H}-NMR** (162 MHz, CD_3CN): δ = -1.10 (s, 2P), -1.77 (s, 2P), -2.54 (s, 1P), -11.03 (d, J = 14.1 Hz, 1P), -12.41 ppm (d, J = 14.4 Hz, 1P). **^{31}P-NMR** (162 MHz, CD_3CN): δ = -1.09 (m, 2P), -1.77 (m, 2P), -2.54 (m, 1P), -11.05 (dtt, J = 14.1, 7.4, 7.1 Hz, 1P), -12.41 ppm (dd, J = 13.4, 13.4 Hz, 1P). **^{13}C-NMR** (101 MHz, CD_3CN): δ = 170.4, 151.7, 138.5 – 136.7 (m), 135.3, 130.1, 129.7 – 128.3 (m), 122.8, 76.5 (m), 74.6 (m), 70.9 – 69.9 (m), 69.3, 21.2. **HRMS (ESI):** *m/z* calcd for $C_{94}H_{94}O_{21}{}^{18}O_{10}P_7$ [M – H$^+$]$^-$: 1956.4405, found: 1956.4341.

[$^{18}O_{10}$]5-InsP$_7$

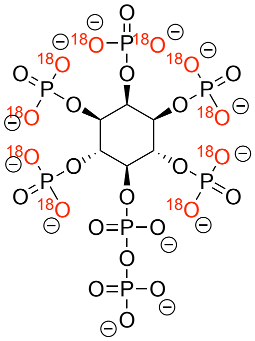

[$^{18}O_{10}$]Bn$_{10}$-5-AB$_2$-InsP$_7$ (43 mg, 21 µmol, 1.0 eq.) was dissolved in a mixture of *t*BuOH and H_2O (4:1, 3 ml). $NaHCO_3$ (14 mg, 167 µmol, 8.0 eq.) and Pd/C (10 wt.% loading, 334 mg, 313 µmol, 15.0 eq.) were added. The mixture was stirred in an autoclave under H_2 pressure (30 bar) for 4.5 h. H_2O (3 ml) was added and further stirred under H_2 pressure overnight. The catalyst was filtered off and washed with water. The combined aq. solutions were diluted with water (to 30 ml) and washed with CH_2Cl_2 (3 x 10 ml). Lyophilization gave the product [19 mg, 20.5 µmol, 98%] as a white solid.

Due to the breadth of the signals, no multiplicities can be assigned.

^{1}H-NMR (400 MHz, D_2O): δ = 4.79 (1H), 4.47 (2H), 4.26 (1H), 4.17 ppm (2H). **^{31}P{^{1}H}-NMR** (162 MHz, D_2O): δ = 0.60 – -0.39 (5P), -9.80 (1P), -10.68 ppm (1P). **^{31}P-NMR** (162 MHz, D_2O): δ = 0.57 – -0.37 (5P), -9.85 (1P), -10.65 ppm (1P). **^{13}C-NMR** (101 MHz, D_2O): δ = 77.7, 75.7, 73.3 ppm. **HRMS (ESI):** *m/z* calcd for $C_6H_{17}O_{17}{}^{18}O_{10}P_7$ [M – 2 H$^+$]$^{2-}$: 378.9278, found: 378.9277. **Isotope enrichment** (CE-ESI-QTOF, [M^{2-}]): M_0 + 20 (378.9278) = 86%, M_0 + 18 (377.9257) = 14%.

[$^{18}O_{12}$]5-InsP$_7$

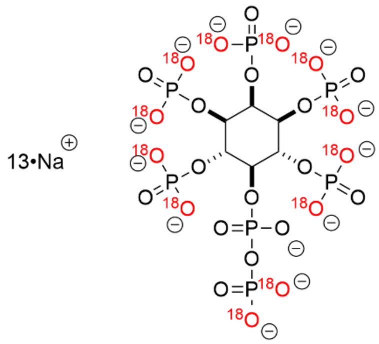

Chemical Formula: $C_6H_6O_{15}{}^{18}O_{12}P_7{}^{13-}$
Exact Mass: 750.7841

[$^{18}O_{12}$]Bn$_{12}$-5-InsP$_7$ (10 mg, 5.4 µmol, 1.0 eq.) was dissolved in a mixture of *t*BuOH and H_2O (4:1, 1 ml). $NaHCO_3$ (4.0 mg, 48 µmol, 8.9 eq.) and Pd/C (10 wt.% loading, 50 mg, 47 µmol, 10.0 eq.) were added. The mixture was stirred in an autoclave under H_2 pressure (30 bar) for 3.5 h. H_2O (0.5 ml) was added and further stirred under H_2 pressure for 1h. The catalyst was filtered off and washed with water. The combined aq. solutions were lyophilized to yield the product [5 mg, 4.68 µmol, 87%] as a white solid.

^{1}H-NMR (400 MHz, D_2O): δ = 4.53 (wH), 4.46 (4H). **^{31}P{^{1}H}-NMR** (162 MHz, D_2O): δ = 4.50 (1P), 3.24 (2P), 2.90(2P), -4.89 (1P), -9.74 ppm (1P). **^{13}C-NMR** (101 MHz, D_2O): δ = 73.8, 73.8, 72.5 ppm. **HRMS (ESI):** *m/z* calcd for $C_6H_{19}O_{15}{}^{18}O_{12}P_7$ [M – 2 H$^+$]$^{2-}$: 380.9320, found: 380.9321. **Isotope enrichment** (CE-ESI-QTOF, [M^{2-}]): M_0 + 24 (380.9320) = 68%, M_0 + 22 (379.9299) = 32%.

7. NMR-spectra

5-Fm₂-InsP₁

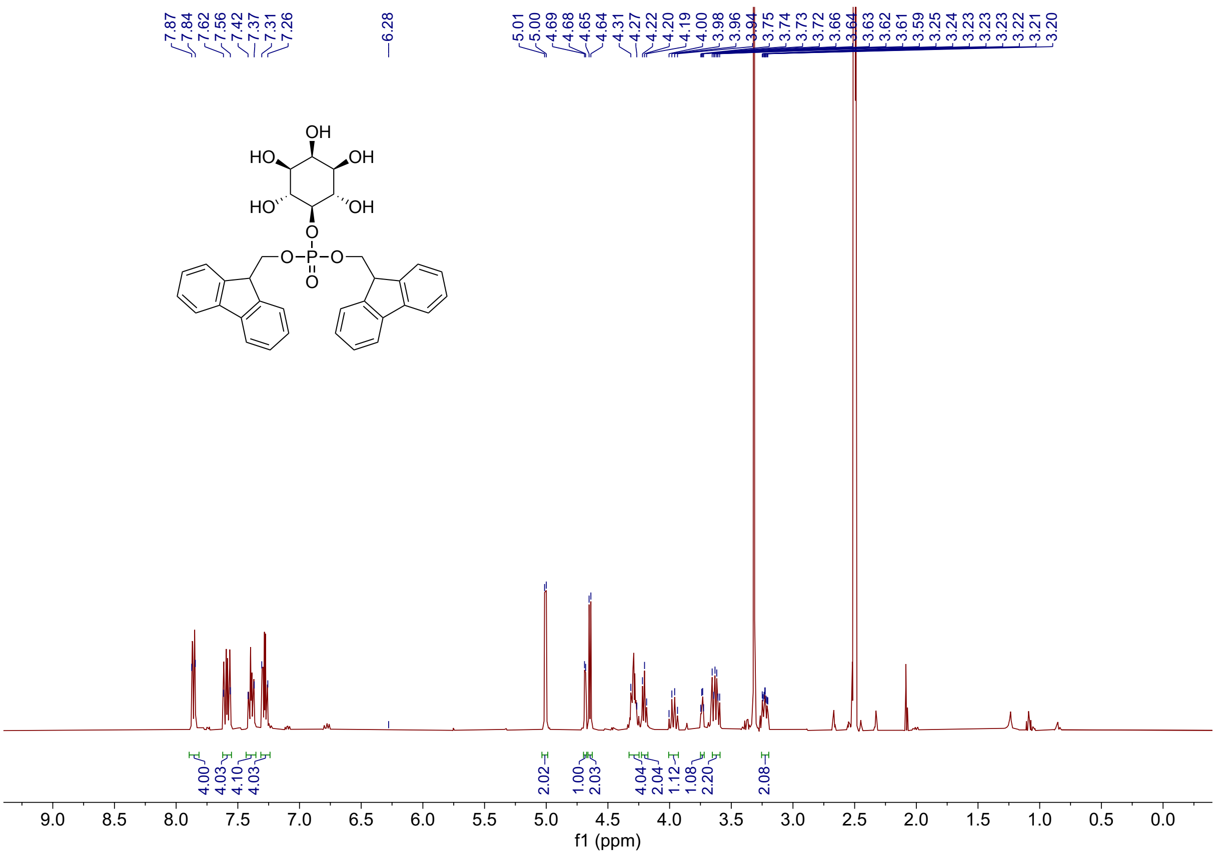

^{1}H-NMR (400 MHz, DMSO-d$_6$) for compound 5-Fm$_2$-InsP$_1$

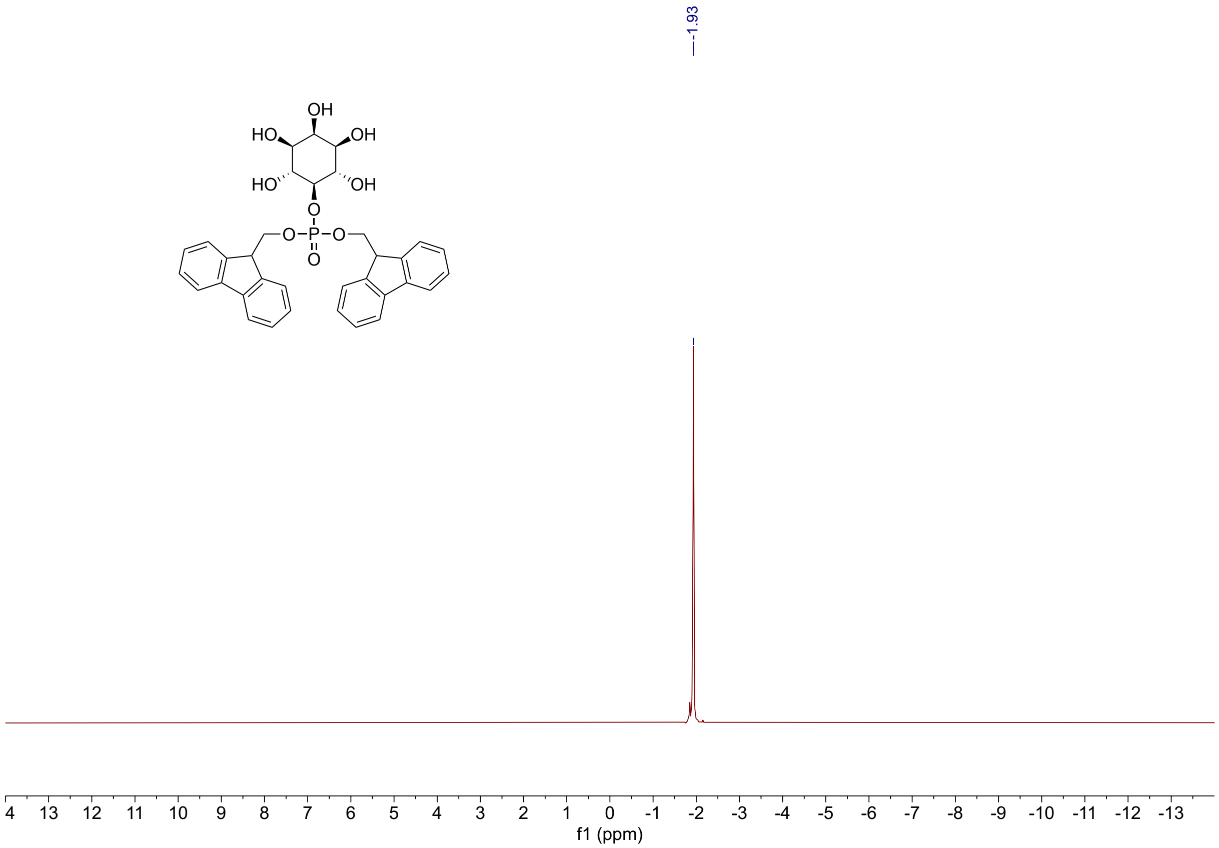

^{31}**P{^{1}H}-NMR** (162 MHz, DMSO-d_6) for compound 5-Fm_2-$InsP_1$

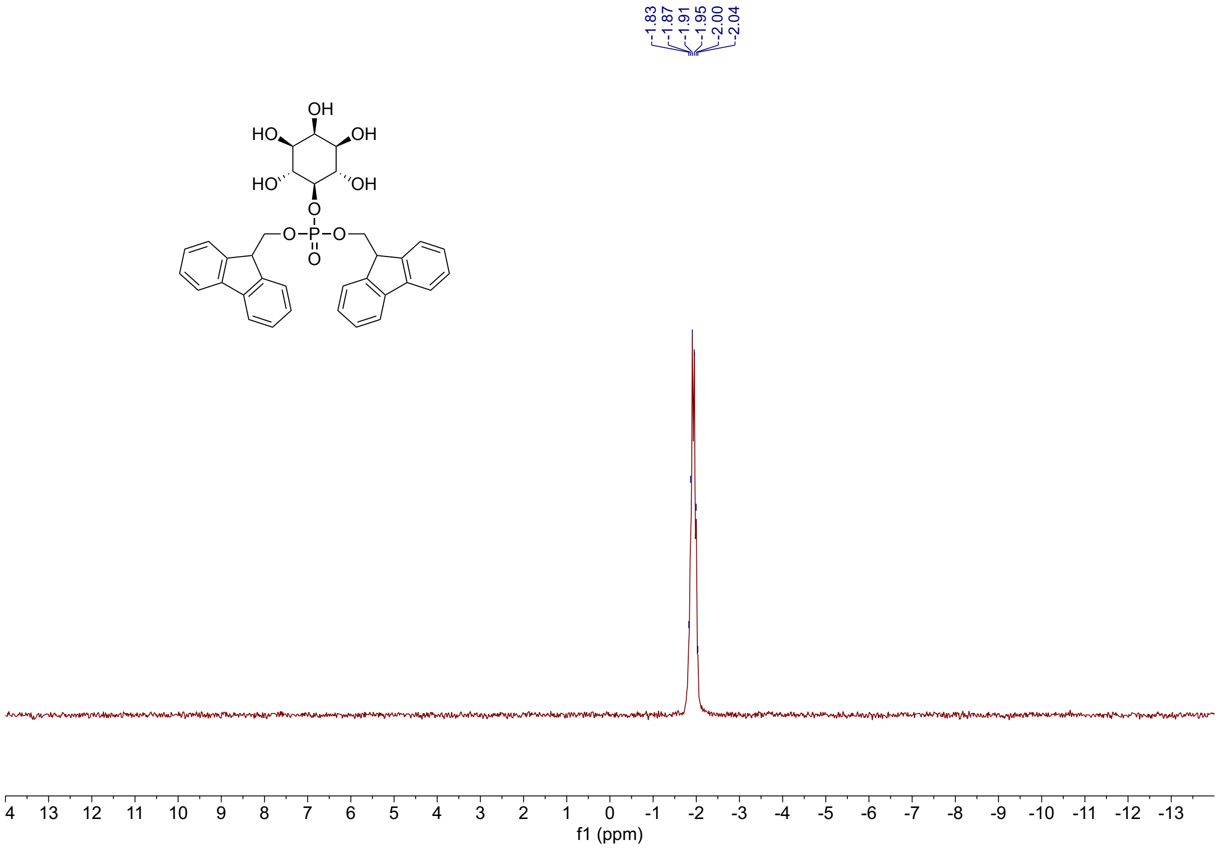

^{31}P-NMR (162 MHz, DMSO-d_6) for compound 5-Fm_2-$InsP_1$

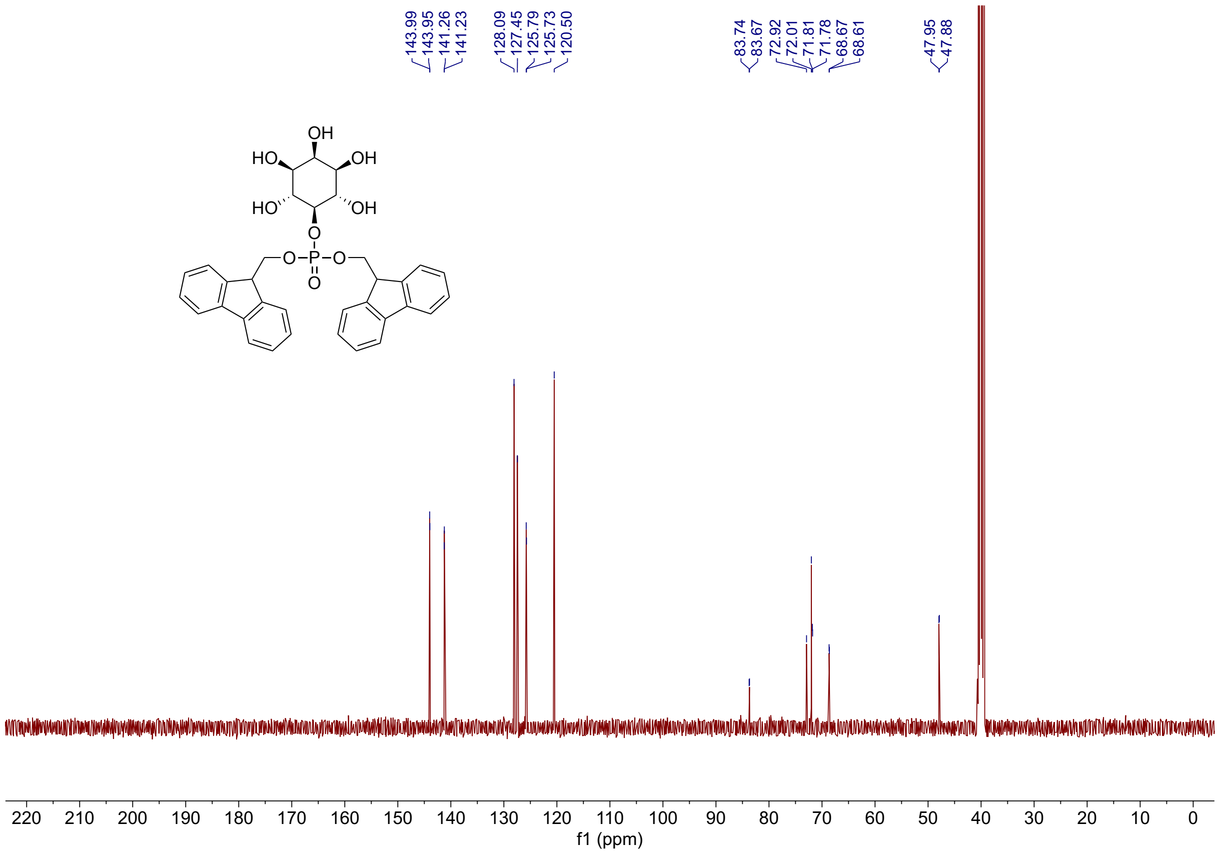

^{13}C-NMR (101 MHz, DMSO-d_6) for compound 5-Fm_2-$InsP_1$

[$^{18}O_{10}$]Bn$_{10}$-5-Fm$_2$-InsP$_6$

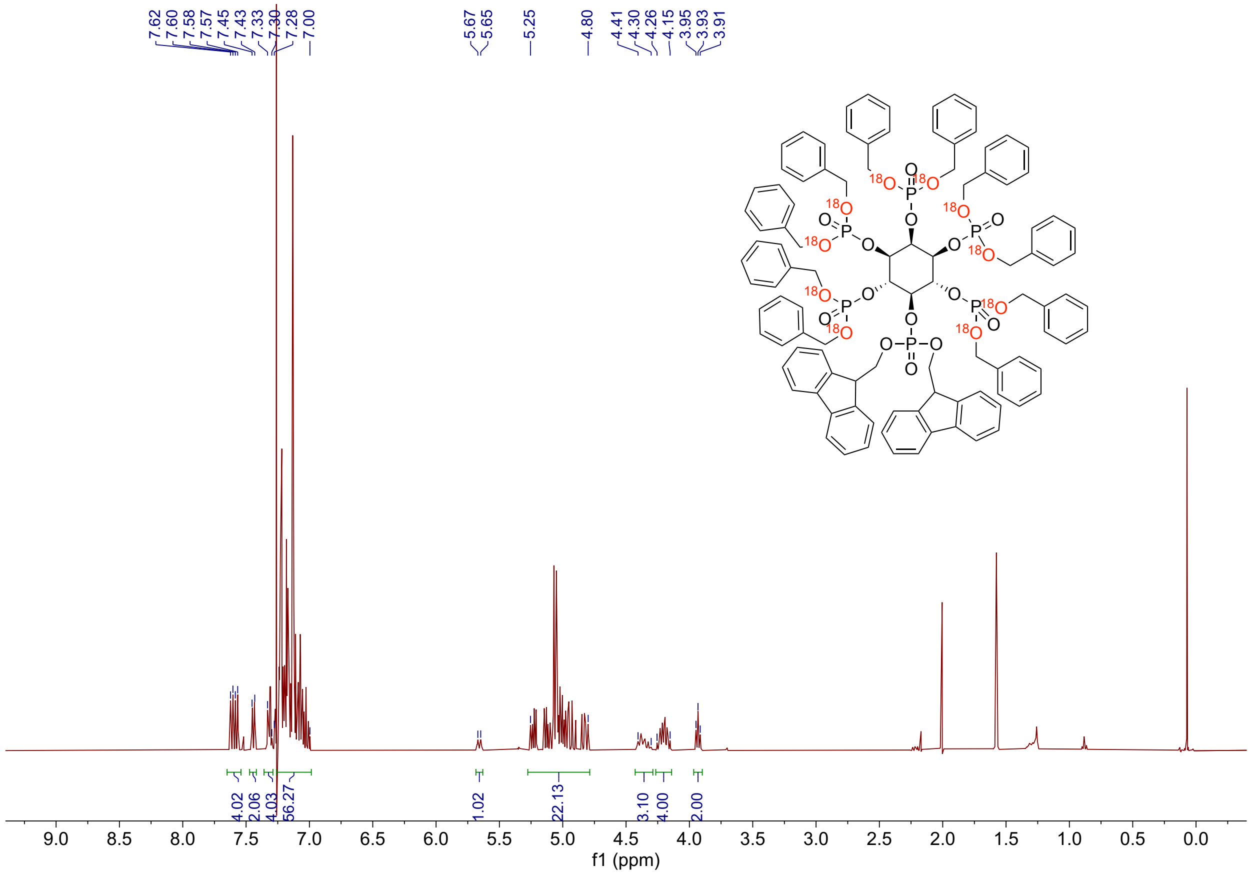

^{1}H-NMR (400 MHz, D_2O) for compound [$^{18}O_{10}$]Bn$_{10}$-5-Fm$_2$-InsP$_6$

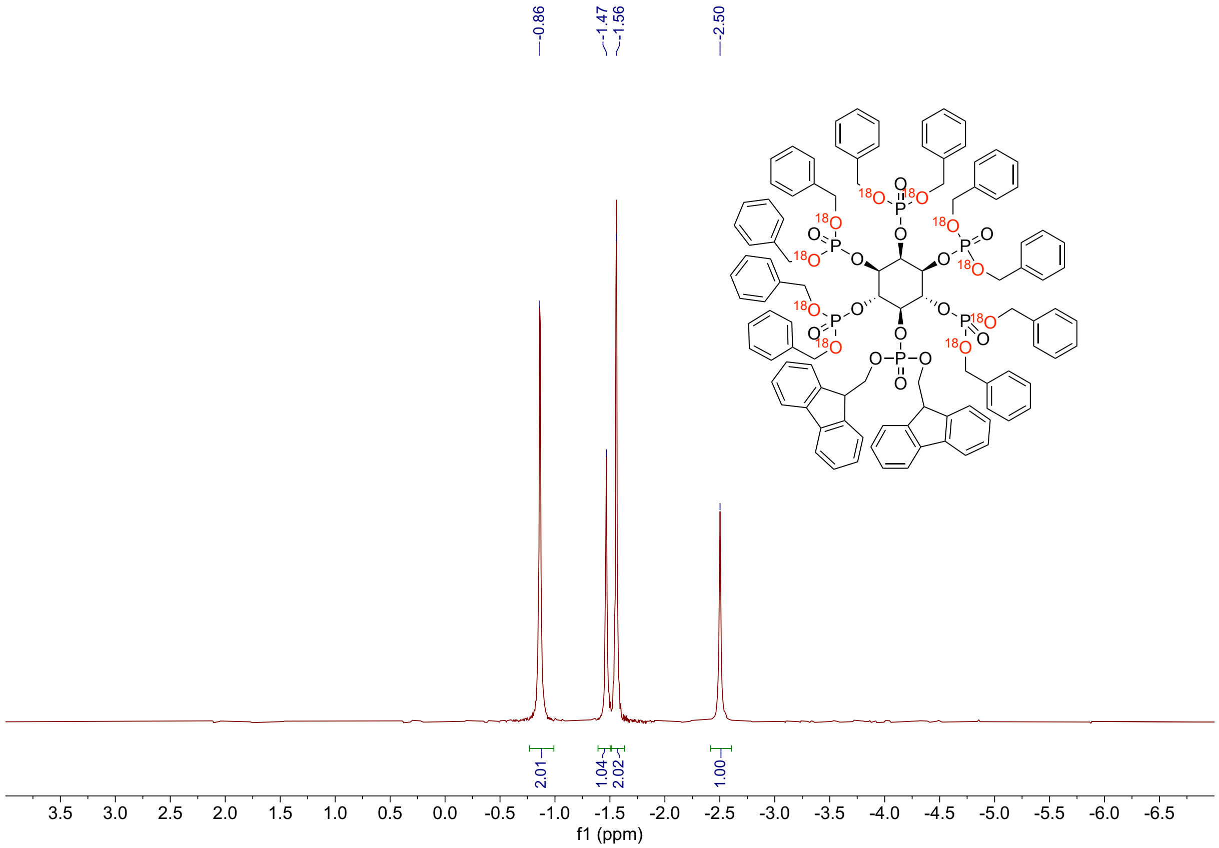

^{31}P{^{1}H}-NMR (162 MHz, D_2O) for compound [$^{18}O_{10}$]Bn_{10}-5-Fm_2-$InsP_6$

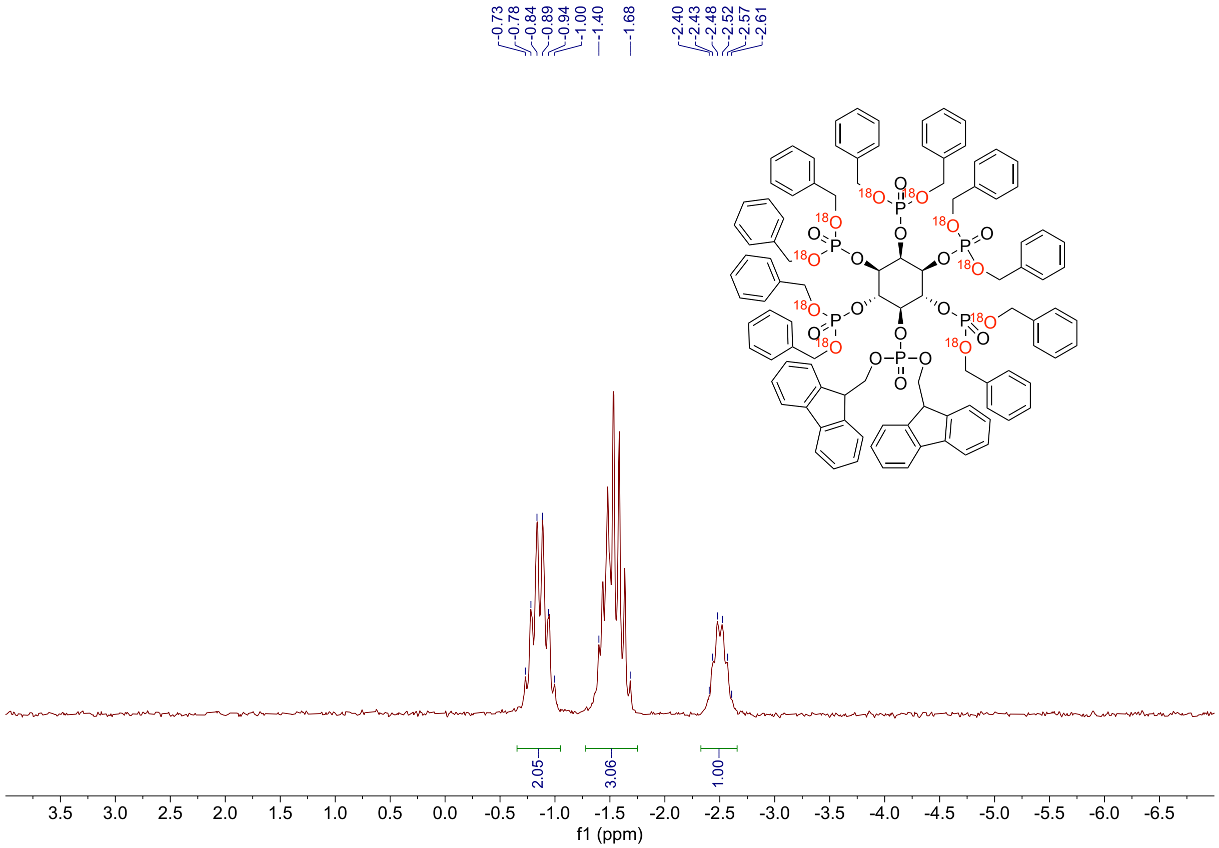

^{31}**P-NMR** (162 MHz, D_2O) for compound [$^{18}O_{10}$]Bn_{10}-5-Fm_2-$InsP_6$

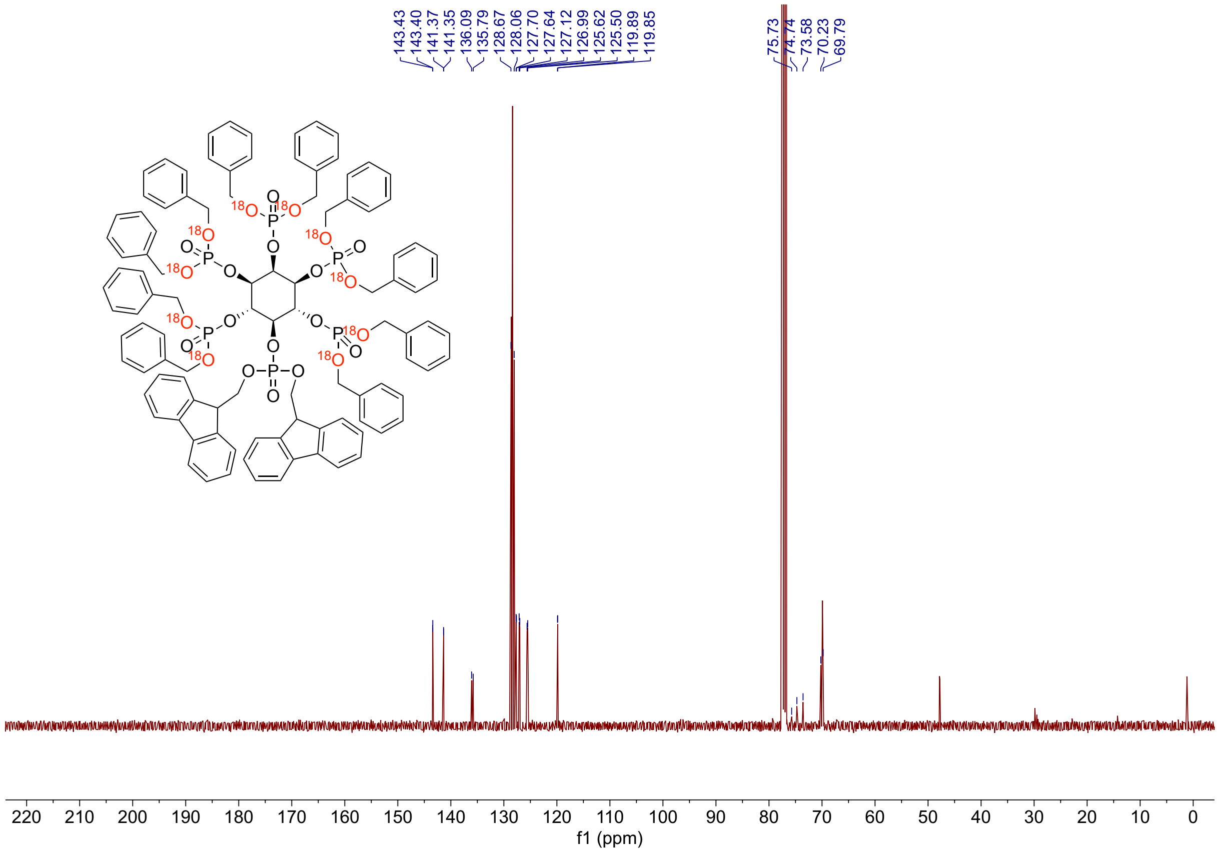

^{13}C-NMR (101 MHz, D_2O) for compound [$^{18}O_{10}$]Bn_{10}-5-Fm_2-$InsP_6$

[$^{18}O_{10}$]Bn$_{10}$-5-AB$_2$-InsP$_7$

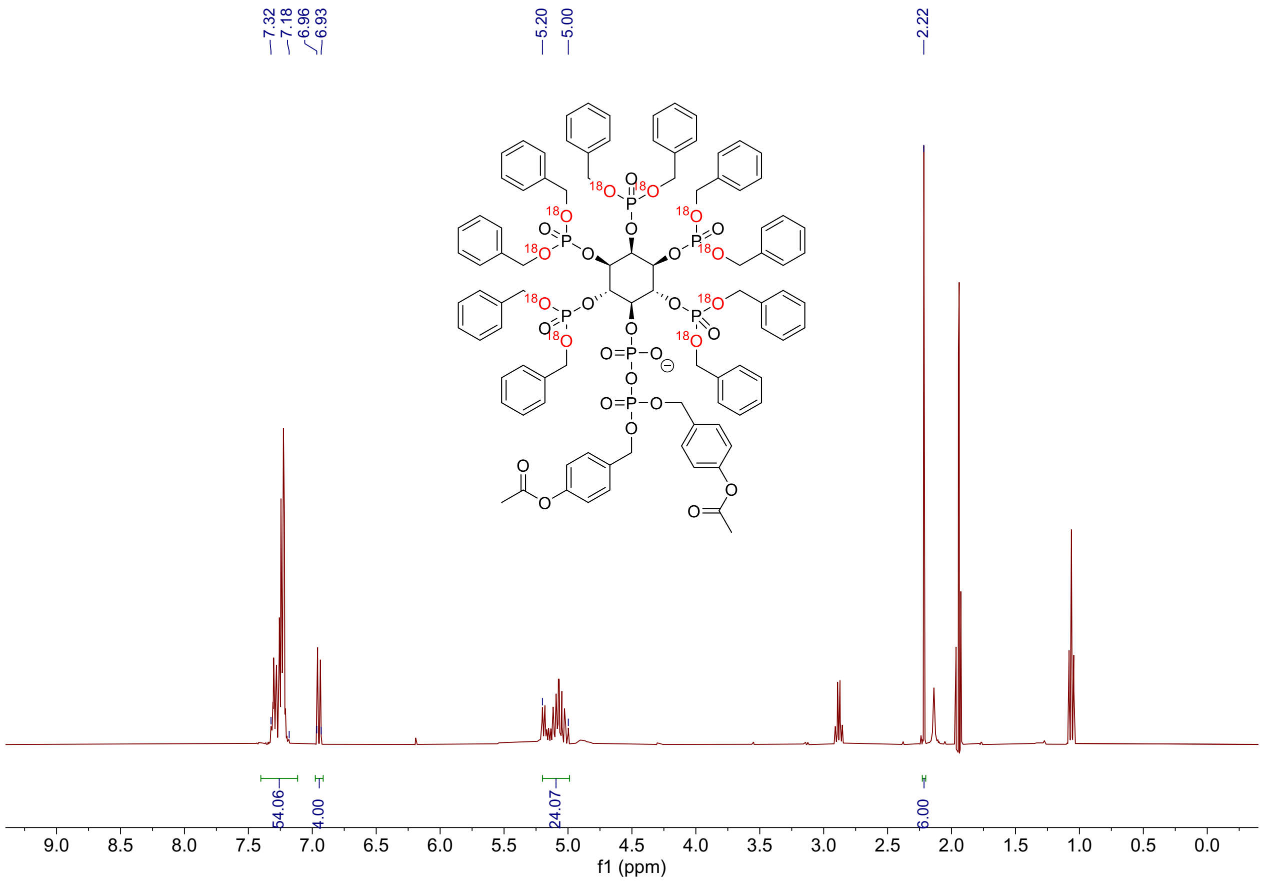

^{1}H-NMR (400 MHz, CD_3CN) for compound [$^{18}O_{10}$]Bn$_{10}$-5-AB$_2$-InsP$_7$

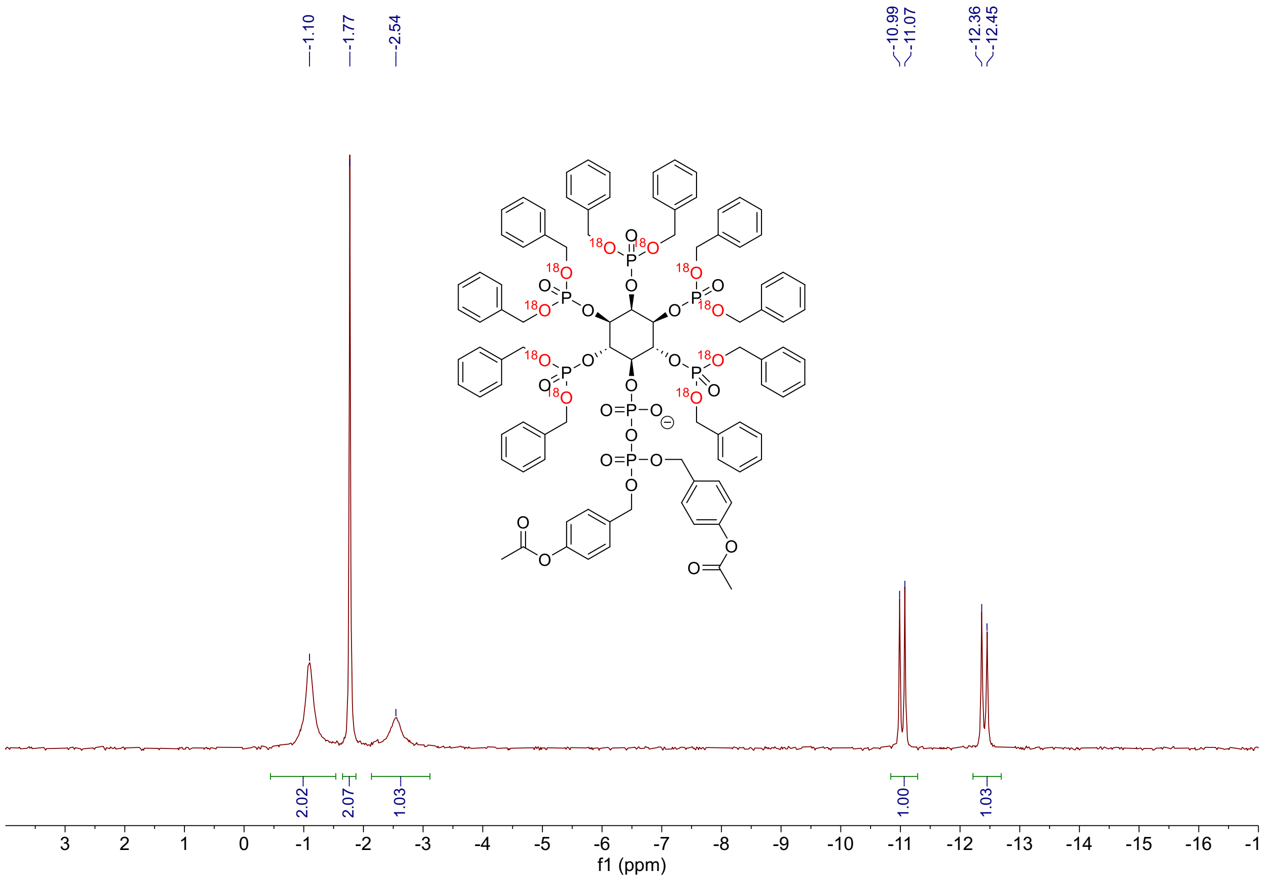

^{31}P{^{1}H}-NMR (162 MHz, CD_3CN) for compound [$^{18}O_{10}$]Bn_{10}-5-AB_2-$InsP_7$

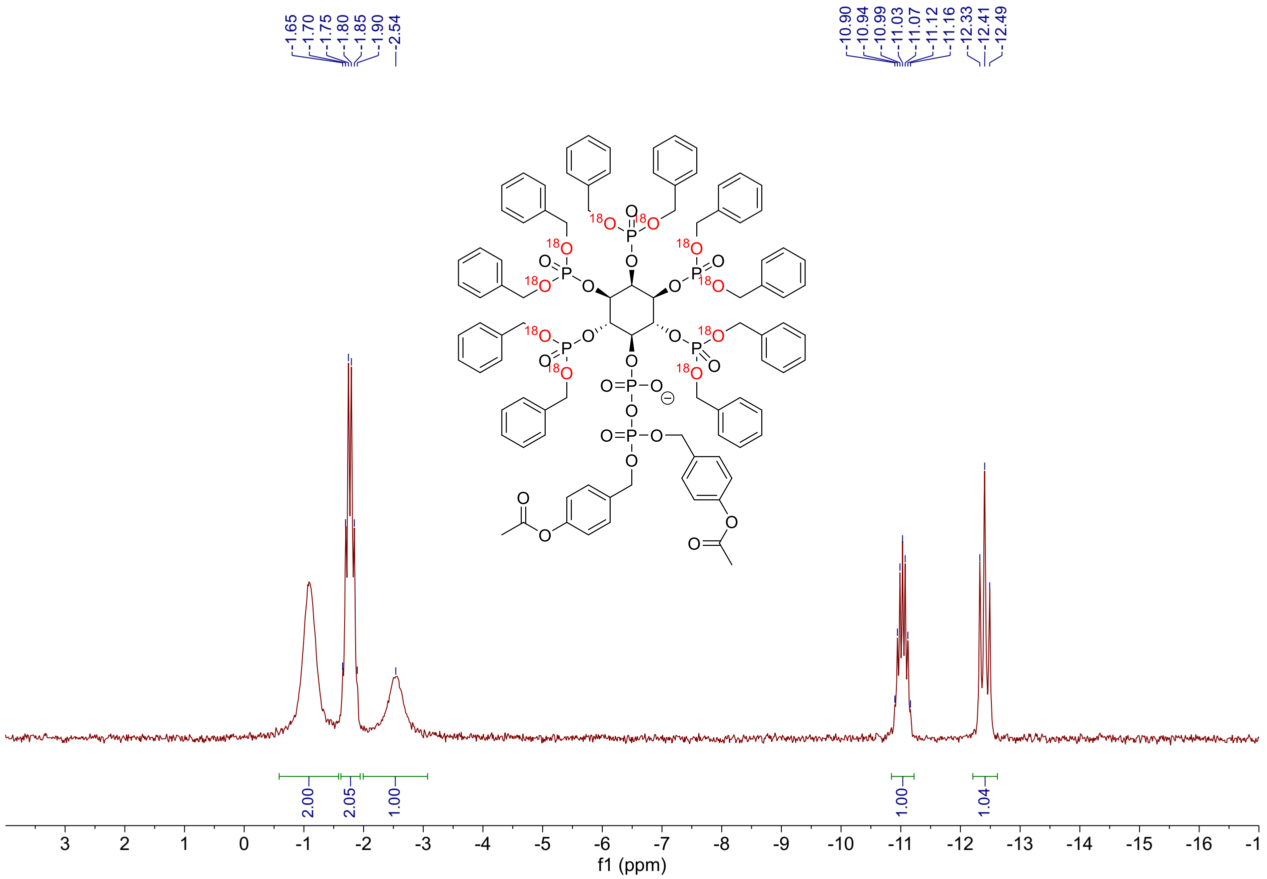

^{31}**P-NMR** (162 MHz, CD_3CN) for compound [$^{18}O_{10}$]Bn_{10}-5-AB_2-$InsP_7$

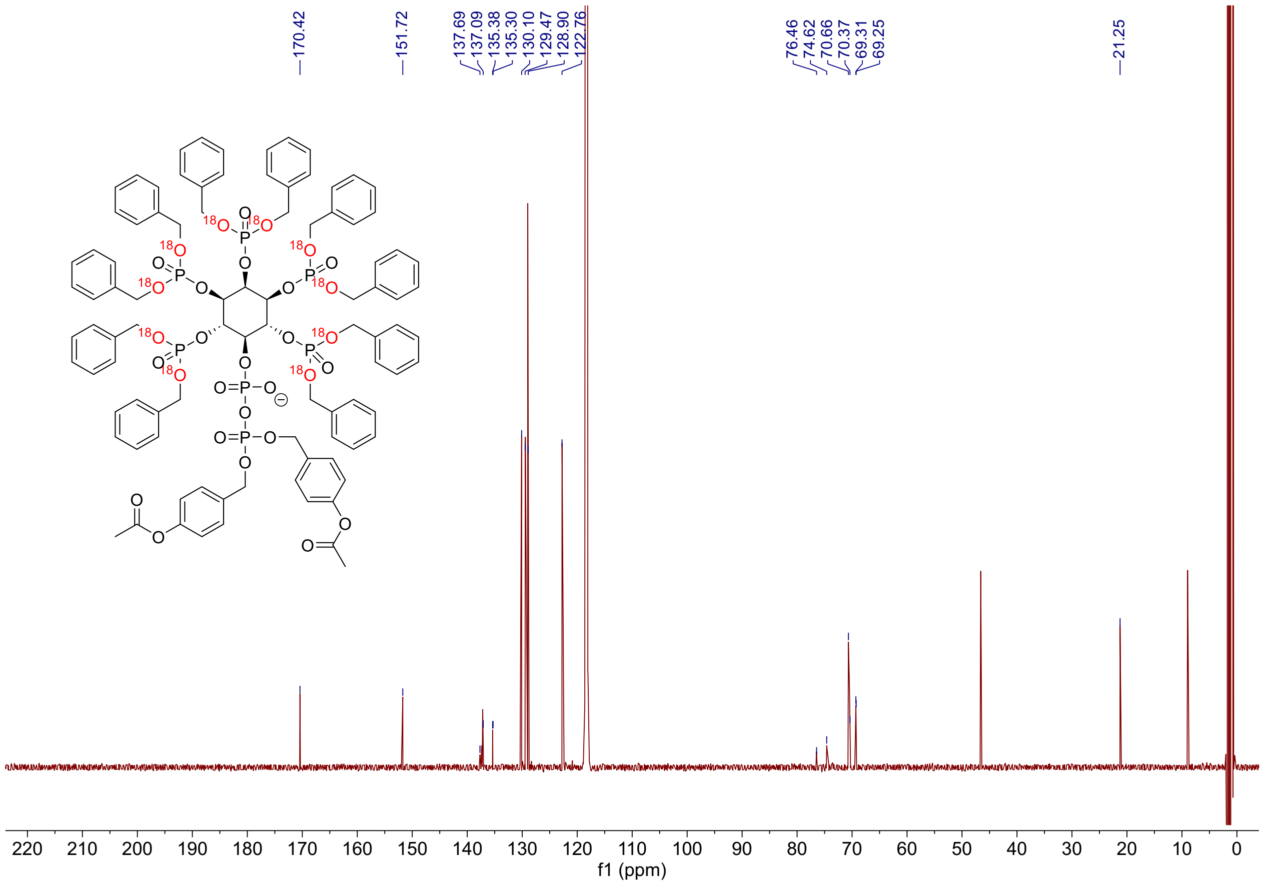

^{13}C-NMR (101 MHz, CD_3CN) for compound [$^{18}O_{10}$]Bn_{10}-5-AB_2-$InsP_7$

[$^{18}O_{10}$]5-InsP$_7$

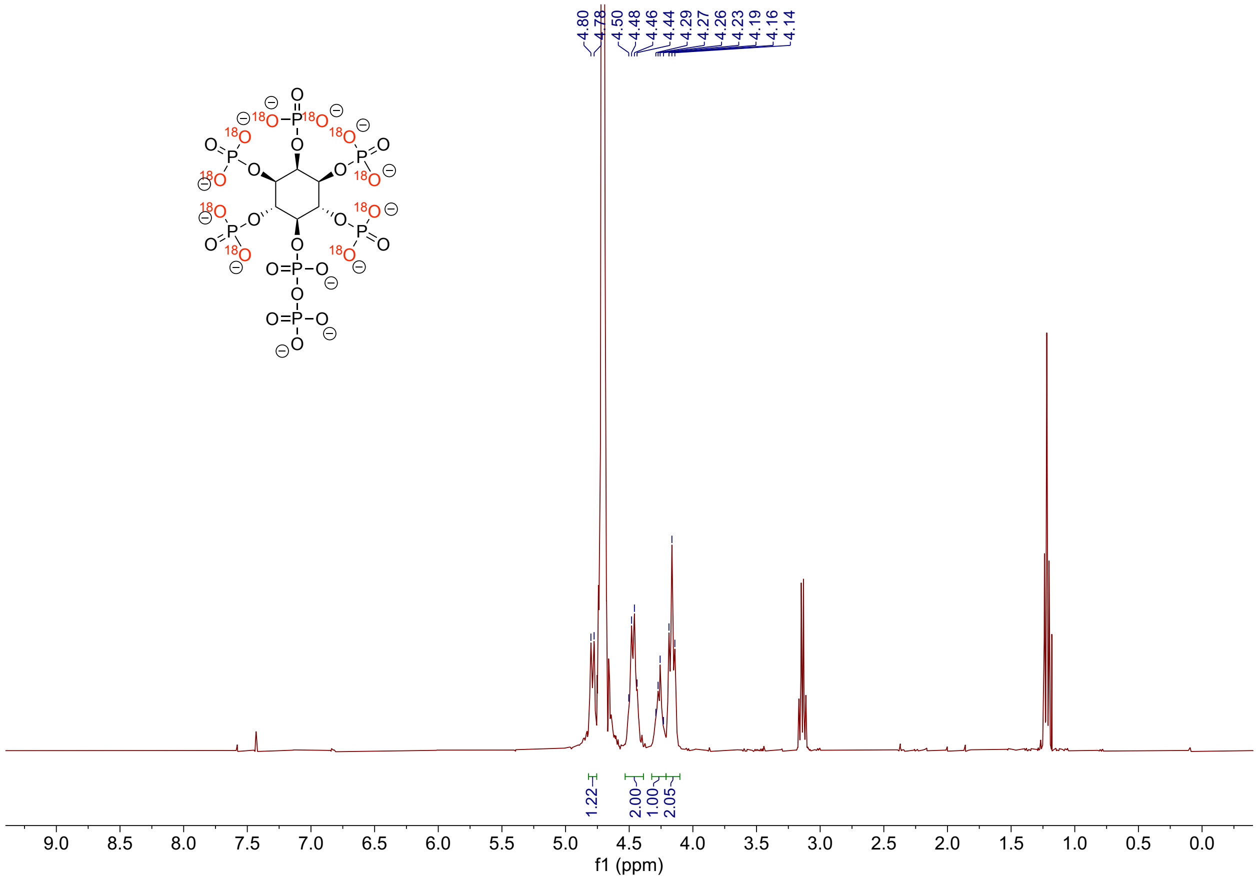

^{1}H-NMR (400 MHz, D_2O) for compound [$^{18}O_{10}$]5-InsP$_7$

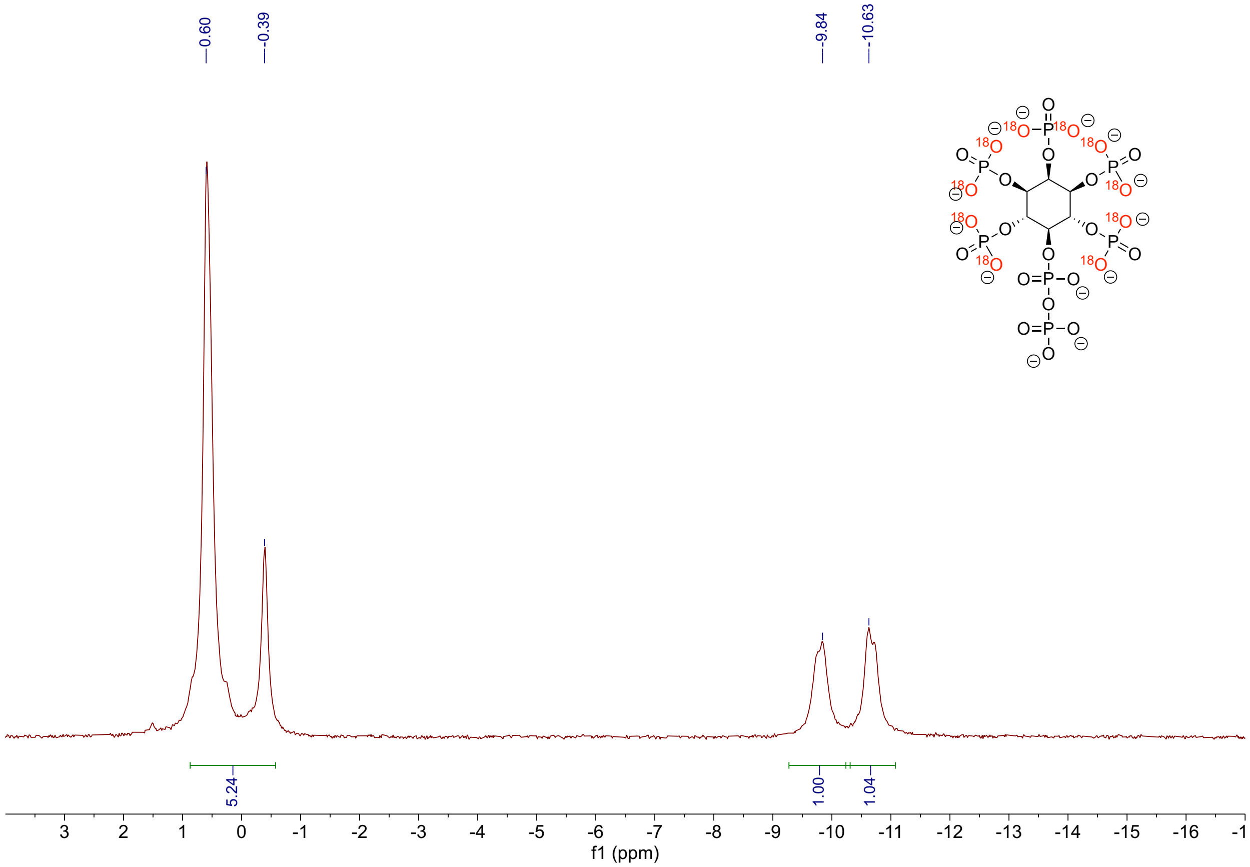

^{31}P{^{1}H}-NMR (162 MHz, D_2O) for compound [$^{18}O_{10}$]5-$InsP_7$

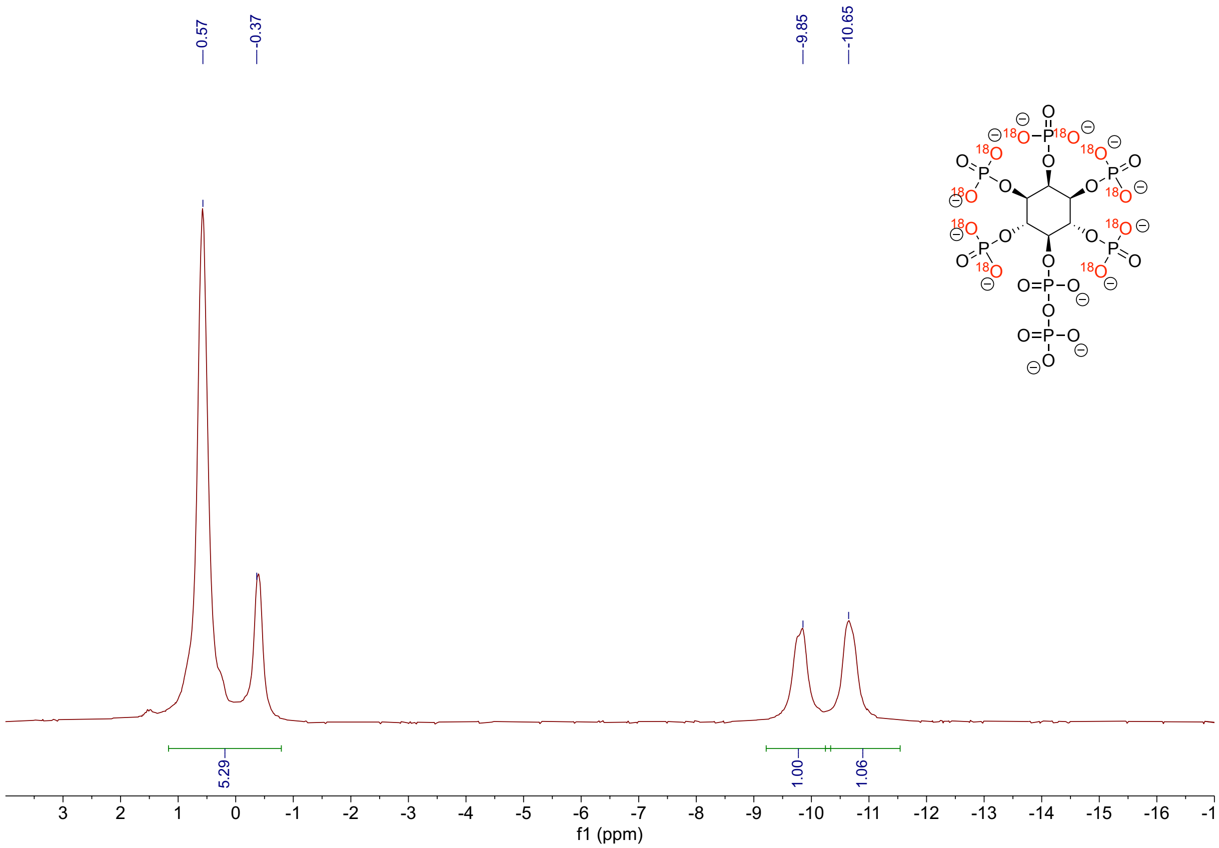

^{31}P-NMR (162 MHz, D_2O) for compound [$^{18}O_{10}$]5-InsP$_7$

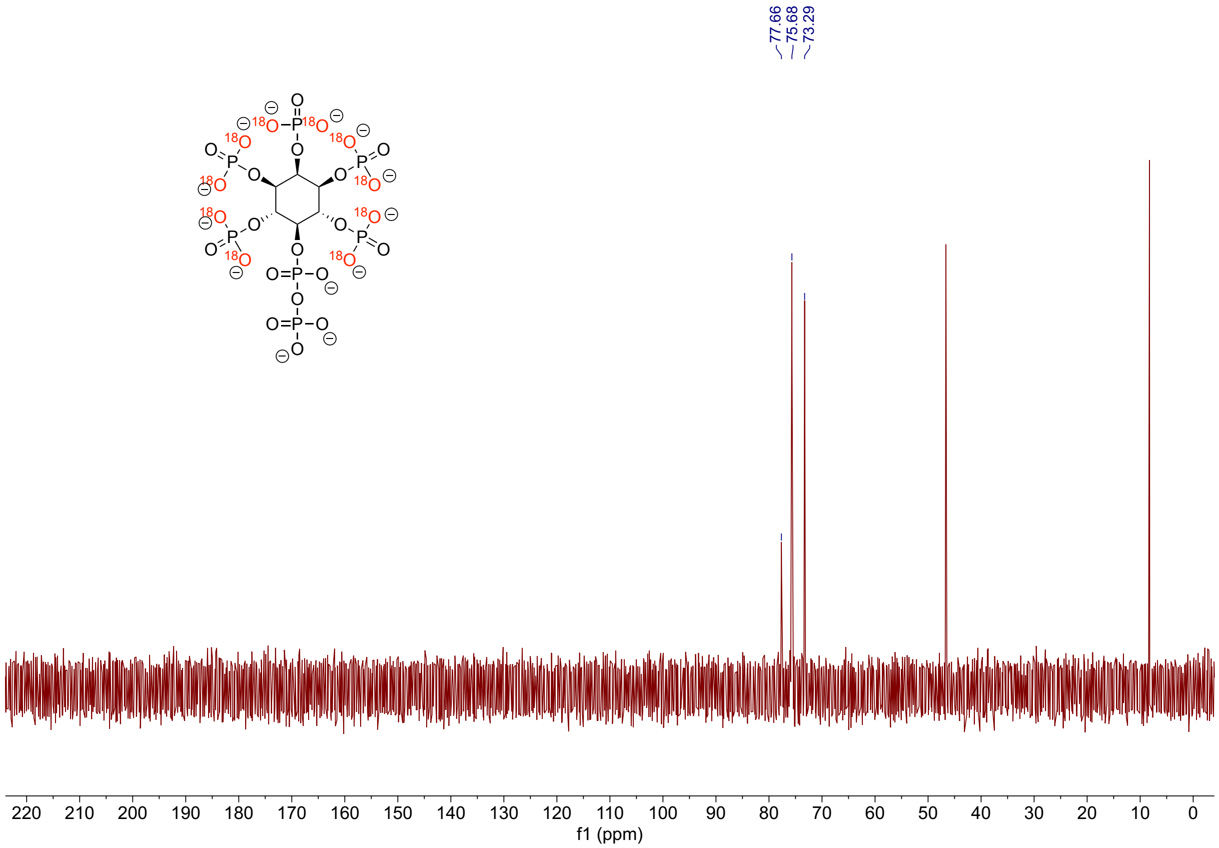

^{13}C-NMR (101 MHz, D_2O) for compound [$^{18}O_{10}$]5-$InsP_7$

[$^{18}O_{12}$]5-InsP$_7$

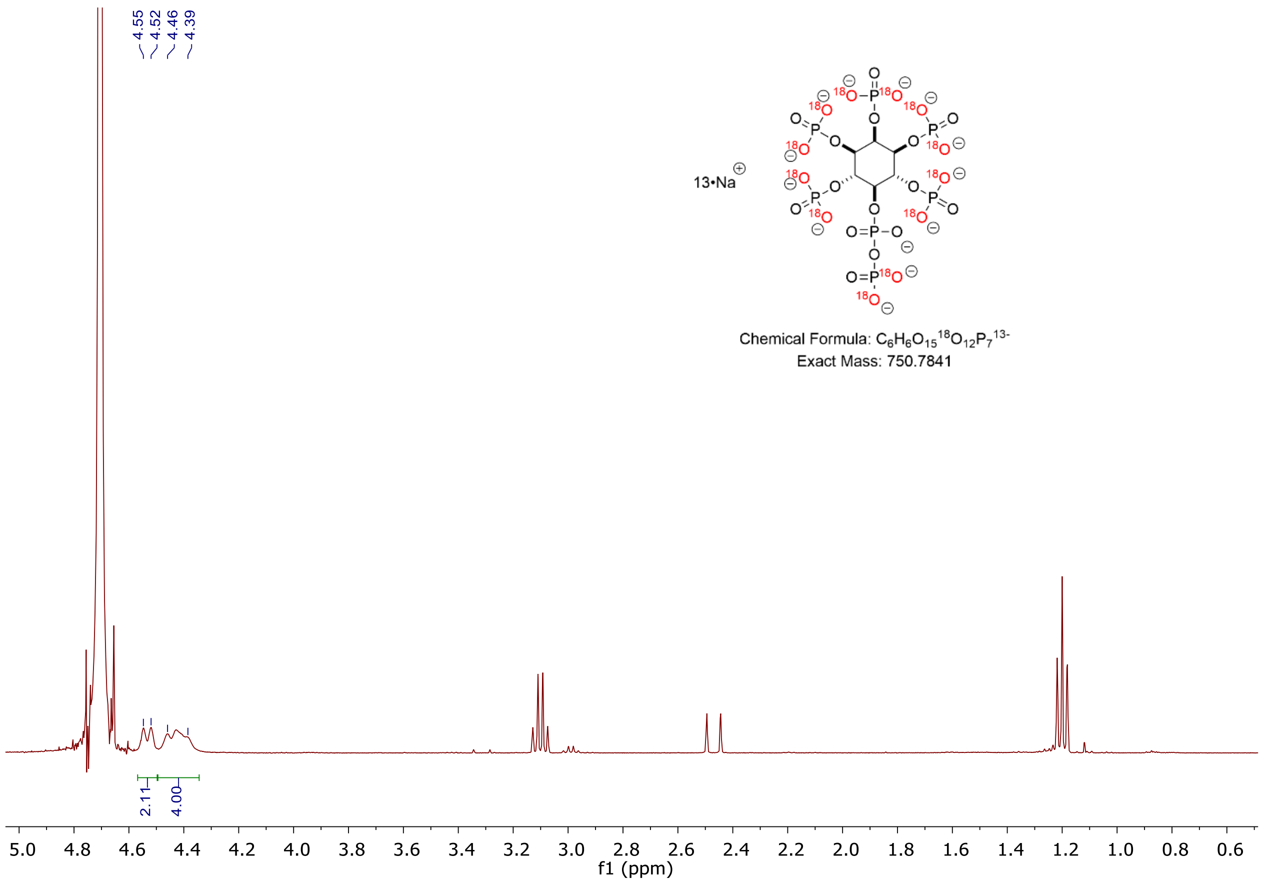

^{1}H-NMR (400 MHz, D_2O) for compound [$^{18}O_{12}$]5-InsP$_7$

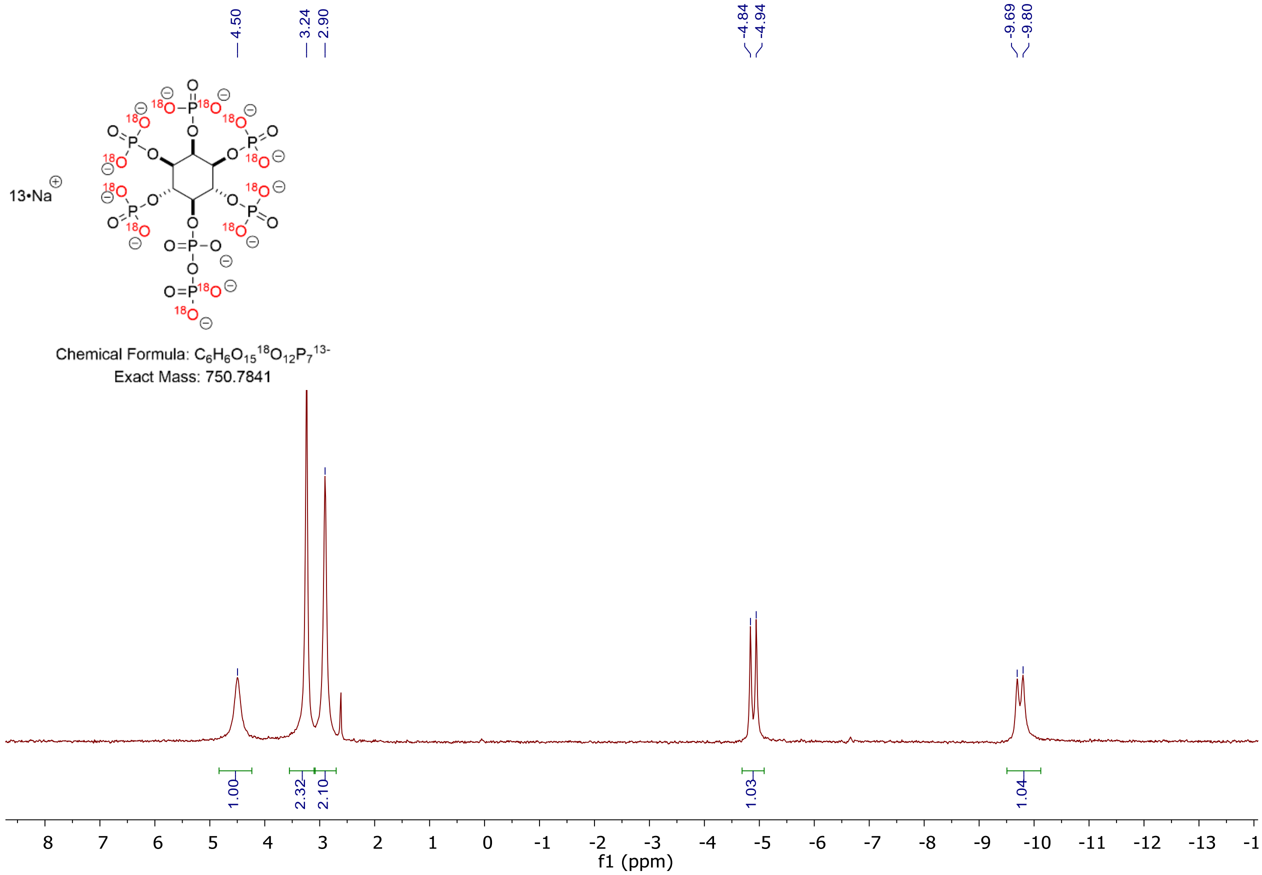

$^{31}P\{^{1}H\}$-NMR (162 MHz, D_2O) for compound $[^{18}O_{12}]$5-InsP$_7$

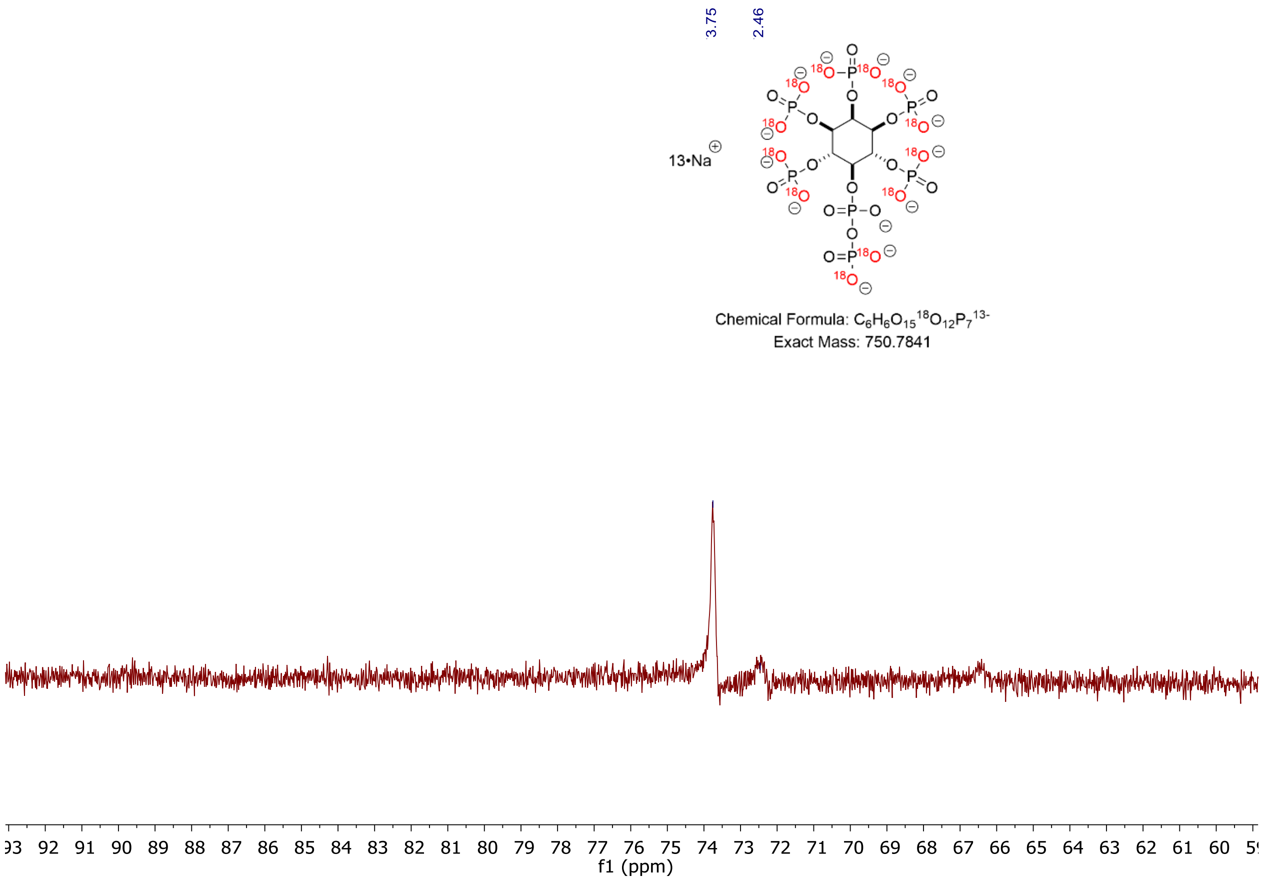

^{13}C-NMR (101 MHz, D_2O) for compound [$^{18}O_{12}$]5-InsP_7
